# Tranexamic acid for the prevention of postpartum bleeding in women with anaemia: study protocol for an international, randomised, double-blind, placebo-controlled trial

**DOI:** 10.1186/s13063-018-3081-x

**Published:** 2018-12-29

**Authors:** Katharine Ker, Ian Roberts, Rizwana Chaudhri, Bukola Fawole, Danielle Beaumont, Eni Balogun, Danielle Prowse, Tracey Pepple, Kiran Javaid, Aasia Kayani, Sabaratnam Arulkumaran, Imelda Bates, Haleema Shakur-Still, on behalf of the WOMAN-2 trial collaborators

**Affiliations:** 1 Clinical Trials Unit, London School of Hygiene and Tropical Medicine, London, UK; 2 Rawalpindi Medical College, Rawalpindi, Pakistan; 3 University College Hospital, Ibadan, Nigeria; 4 Pakistan Regional Trials Coordinating Centre, Rawalpindi Medical College, Rawalpindi,, Pakistan; 5 St. George’s, University of London, London, UK; 6 Liverpool School of Tropical Medicine, Liverpool, UK

**Keywords:** Antifibrinolytic, Clinical trial, Tranexamic acid, Postpartum haemorrhage, Maternal anaemia

## Abstract

**Background:**

Postpartum haemorrhage (PPH) is responsible for about 100,000 maternal deaths every year, most of which occur in low- and middle-income countries. Tranexamic acid (TXA) reduces bleeding by inhibiting the enzymatic breakdown of fibrin blood clots. TXA decreases blood loss in surgery and reduces death due to bleeding after trauma. When given within 3 h of birth, TXA reduces deaths due to bleeding in women with PPH. However, for many women, treatment of PPH is too late to prevent death. Over one third of pregnant women in the world are anaemic and many are severely anaemic. These women have an increased risk of PPH and suffer more severe outcomes if PPH occurs. There is an urgent need to identify a safe and effective way to reduce postpartum bleeding in anaemic women.

**Methods/design:**

The WOMAN-2 trial is an international, multicentre, randomised, double-blind, placebo-controlled trial to quantify the effects of TXA on postpartum bleeding in women with moderate or severe anaemia. Ten thousand women with moderate or severe anaemia who have given birth vaginally will be randomised to receive 1 g of TXA or matching placebo by intravenous injection immediately (within 15 min) after the umbilical cord is cut or clamped. The primary outcome is the proportion of women with a clinical diagnosis of primary PPH. The cause of PPH will be described. Data on maternal health and wellbeing, maternal blood loss and its consequences, and other health outcomes will be collected as secondary outcomes. The main analyses will be on an ‘intention-to-treat’ basis, irrespective of whether the allocated treatment was received. Results will be presented as appropriate effect estimates with a measure of precision (95% confidence intervals). Subgroup analyses will be based on the severity of anaemia (moderate versus severe) and type of labour (induced or augmented versus spontaneous). A study with 10,000 patients will have over 90% power to detect a 25% relative reduction from 10 to 7.5% in PPH. The trial will be conducted in hospitals in Africa and Asia.

**Discussion:**

The WOMAN-2 trial should provide reliable evidence for the effects of TXA for preventing postpartum bleeding in women with anaemia.

**Trial registration:**

ISRCTN, ISRCTN62396133. Registered on 7 December 2017;

ClincalTrials.gov, ID: NCT03475342. Registered on 23 March 2018.

**Electronic supplementary material:**

The online version of this article (10.1186/s13063-018-3081-x) contains supplementary material, which is available to authorized users.

## Background

Postpartum haemorrhage (PPH) is a leading cause of maternal mortality and morbidity. PPH follows 6 to 10% of all births and accounts for around 100,000 maternal deaths every year [1–3]. Ninety-nine percent of deaths are in low- and middle-income countries (LMICs) [4]. Many women who survive experience severe morbidity. Some women need surgery to control the bleeding (e.g. exploratory laparotomy, uterine artery ligation, brace sutures) and many require a hysterectomy, thus removing the possibility of having more children. Severe morbidity due to PPH interferes with breastfeeding and bonding [5]. PPH is a frightening experience and some women develop post-traumatic stress disorder [6].

Many women with PPH are given a blood transfusion. However, blood is a scarce and costly resource in LMICs and access to safe blood is limited. The blood donation rate in Africa is 5 per 1000 population compared to 47 per 1000 population in the USA and it is estimated that 35 of the 40 sub-Saharan countries collect less than half of the donor blood required to meet their population needs [7]. Even when blood is available, because of problems with screening, recipients are at risk of blood-borne infections and adverse transfusion reactions are common.

Anaemia is a cause and consequence of PPH. A cohort study in Assam, India found that women with moderate or severe anaemia had a greatly increased risk of PPH [8]. Women with moderate anaemia had a 50% increased risk of PPH, whereas those with severe anaemia had a tenfold increased risk. The reasons for the increased risk is unclear but some researchers think that anaemic women are more susceptible to uterine atony due to impaired oxygen transport to the uterus. Anaemic women experience worse outcomes after PPH. An international survey of 275,000 women found that severe maternal outcomes after PPH were nearly three times more common in anaemic than in non-anaemic women [9]. Even moderate bleeding can be life-threatening in anaemic women. Excessive bleeding after childbirth worsens maternal anaemia, raising the possibility of a vicious circle of bleeding and adverse outcomes. Fatigue due to anaemia limits a mother’s wellbeing and her ability to care for her children [10]. Despite efforts to prevent anaemia, many women labour with low haemoglobin (Hb) levels. Worldwide, over 32 million pregnant women are anaemic, about 800,000 of whom are severely anaemic [11]. The prevalence is highest in countries in central and West Africa as well as in South Asia where about half of pregnant woman are anaemic and it poses a severe public health problem [11, 12]. There is an urgent need to find an effective way to reduce postpartum bleeding in anaemic women.

Tranexamic acid (TXA) is a synthetic analogue of the amino acid lysine, which inhibits fibrinolysis by blocking the lysine binding sites on plasminogen. TXA reduces surgical bleeding and death due to bleeding in trauma patients. The WOMAN trial assessed the effects of TXA in 20,060 women with PPH [13]. TXA significantly reduced death due to bleeding with no adverse effects. When given within 3 h of birth, TXA reduced death due to bleeding by nearly one third (Relative Risk (RR) = 0.69, 95% CI 0.52 to 0.91; *P* = 0.008). However, for many women, treatment is too late to prevent death from PPH. Most PPH deaths occur in the first hours after giving birth and women with anaemia are at increased risk. Whilst there have been some trials of TXA for the prevention of PPH, most have serious flaws and very few collected data on maternal wellbeing. Most of the trials are small, low-quality, single-centre studies and only one was prospectively registered. Many trials were not properly randomised and were imbalanced for key prognostic variables. Furthermore, several of the trial reports contained important errors or inconsistencies and at least two trials did not have ethics approval. There is very little reliable evidence about the effectiveness and safety of TXA for preventing postpartum bleeding, especially in high-risk anaemic women.

The WOMAN-2 trial should determine the effects of TXA in women with moderate or severe anaemia who give birth vaginally. For pregnant women, the World Health Organisation (WHO) defines moderate anaemia as Hb levels of 70–99 g/L and severe anaemia as Hb levels lower than 70 g/L [14]. Women with anaemia are at increased risk of PPH and experience worse outcomes should PPH occur. By including women with moderate or severe anaemia, participating women have the potential to benefit from the trial treatment. Results from clinical trials of TXA in elective surgery show that TXA reduces blood loss by about one third irrespective of baseline blood loss [15]. In other words, TXA treatment seems to move the entire distribution of bleeding towards reduced blood loss. If this is also the case in postpartum anaemic women, then trial participants have the potential to benefit whether or not they experience PPH, since even moderate or mild blood loss can have adverse health consequences in anaemic women.

Around 10,000 women with moderate or severe anaemia giving birth in hospitals primarily in Africa and Asia will be randomly allocated to receive TXA or matching placebo after the umbilical cord is cut or clamped. Although there is no evidence of any adverse effects on the baby, by randomising women after cutting or clamping the umbilical cord, any risk associated with placental transfer of the trial treatment to the baby is removed. The umbilical cord will be cut or clamped in the usual way and the timing will not be affected by the trial. TXA passes into breast milk in very low concentrations and so an antifibrinolytic effect in the baby is highly unlikely.

### How does tranexamic acid prevent excessive blood loss?

The ability to form a blood clot depends on fibrinogen levels. In both trauma and PPH, a low serum fibrinogen is a strong predictor of life-threatening bleeding. Fibrinogen declines rapidly during bleeding due to its consumption in fibrin clot formation. However, fibrinolysis due to the activation of plasmin by tissue plasminogen activator (TPA) worsens fibrinogen depletion by breaking down clots. Tissue- plasminogen-activator-mediated fibrinogenolysis also depletes fibrinogen levels. Early TXA administration has the potential to prevent excessive blood loss by interrupting the vicious circle of fibrinolysis and fibrinogen depletion. Women with anaemia are at increased risk of bleeding soon after delivery. If they can be treated with TXA before their fibrinogen levels fall, severe postpartum bleeding and its consequences may be prevented.

### Rationale for trial

For some women the treatment of PPH is too late to prevent death and severe morbidity. Despite efforts to increase the availability of antenatal care, many women are anaemic at the time of giving birth and blood for transfusion is often unavailable. There is an urgent need to reduce postpartum bleeding and its adverse impacts on mothers, especially in anaemic women in LMICs. Knowing that TXA reduces deaths due to bleeding after PPH provides reason to believe that it might also prevent PPH. However, the evidence to date is insufficient to support the prophylactic use of TXA in routine clinical practice. Most of the available trials of TXA for preventing PPH are small and unreliable, and few collect information on maternal health and wellbeing [16, 17]. One exception is the TRAAP trial [18] which enrolled 4079 women who were giving birth vaginally in French hospitals. Women were randomised to receive 1 g TXA or matching placebo within 2 min after delivery. Although women who received TXA were less likely to experience a blood loss of ≥ 500 mL (the primary endpoint), the difference was not statistically significant (RR = 0.83, 95% CI 0.68 to 1.01; *P* = 0.07). Fewer woman in the TXA group received additional uterotonics (RR = 0.75, 95% 0.61 to 0.92; *P* = 0.006); however, there were no statistically significant differences in transfusion, change in Hb or surgical intervention. The WOMAN-2 trial should provide reliable evidence on the effects of TXA when used to prevent PPH in anaemic women in LMICs. Although there was no increase in thrombotic events with TXA in the WOMAN or TRAPP trials, the administration of TXA to all women who give birth vaginally may be inappropriate. There is an increased risk of venous thrombosis in the postpartum period [19] and maternal anaemia is an established risk factor [20]. Treating all mothers would involve treating all women when only a proportion would benefit. However, in anaemic women the benefits could outweigh any harms so that a trial is justified. Inclusion in the trial will be limited to women giving birth vaginally. For women who give birth by caesarean section, especially for placenta abnormalities, the interval between cord clamping and PPH onset is short, often a matter of minutes, so the potential of TXA to prevent coagulopathy and PPH is limited.

### Safety of tranexamic acid

TXA is a widely used treatment with a good safety profile. Although on pathophysiological grounds we might expect an increased risk of thrombosis with antifibrinolytic drugs, randomised trials including over 50,000 participants show no increased risk. High doses of TXA (doses from 7.5 g up to 20 g) have been associated with seizures in cardiac surgery but there was no increase in seizures in the CRASH-2 or WOMAN trials, which used a 1–2 g dose. TXA passes into breast milk in very low concentrations, approximately 100th of the concentration in maternal blood. An antifibrinolytic effect in the breast-fed infant is highly unlikely at this low concentration [21, 22]. No adverse events (AEs) in breastfed babies were found in the WOMAN trial. Because TXA will be given after cutting or clamping the umbilical cord, there will be no risk of placental transfer to the baby. Nevertheless, we will collect data on nausea, vomiting, diarrhoea, maternal thrombotic events, seizures and thromboembolic events in breastfed babies in all participants as outcomes. These outcome events will not be reported using the AE reporting procedure.

## Objectives

To determine the effects of TXA on postpartum bleeding and other health outcomes in women with moderate or severe anaemia.

## Methods/design

This protocol has been prepared in accordance with the Standard Protocol Items: Recommendations for Interventional Trials (SPIRIT) 2013 Statement (Additional files 1 and 2: Figures S1 and S2) [23].

### Overview

**Fig. 1:**
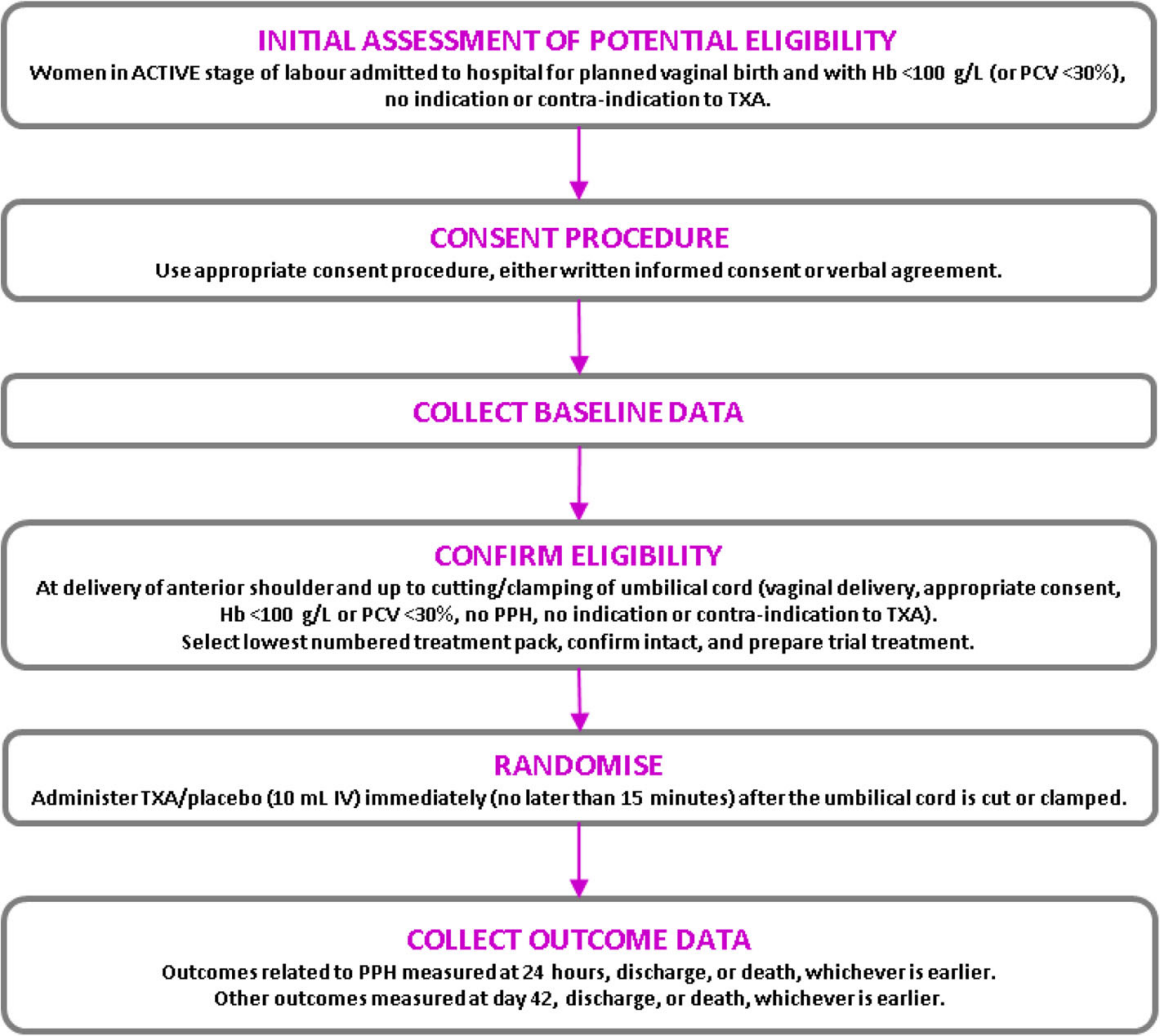
Trial overview

**Fig. 2:**
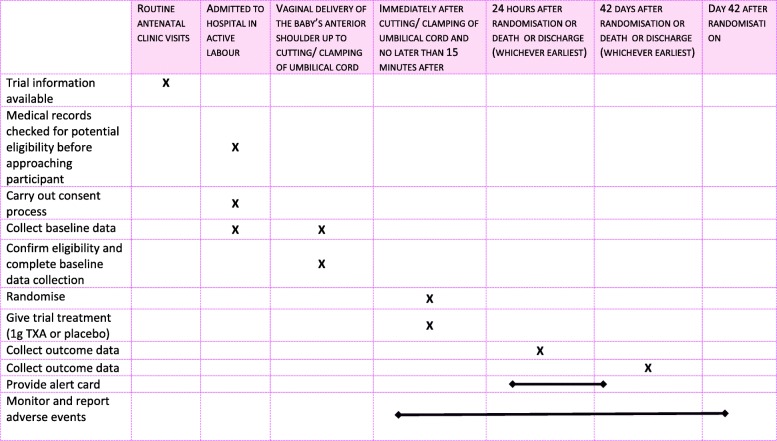
Schedule of procedures, events and assessments

The WOMAN-2 trial is a randomised, parallel-group, double-blind, placebo-controlled trial of the effects of TXA in women with moderate or severe anaemia who are giving birth vaginally. Ten thousand women with moderate or severe anaemia who are giving birth in hospitals will be randomised to receive 1 g of TXA or matching placebo (sodium chloride 0.9%) by intravenous injection immediately and no later than 15 min after the umbilical cord is cut or clamped (Fig. 1). The schedule of enrolment, interventions and assessments according to the SPIRIT 2013 guidelines is presented in Fig. 2.

### Setting

Women from hospitals where anaemia in pregnancy is common, primarily in Africa and Asia, will be enrolled. All participating hospitals will have the facilities to provide comprehensive essential obstetric care as defined by the WHO.

### Number of participants needed

For the purpose of the sample size calculation, a baseline risk of PPH of 10% was assumed. A trial with 10,000 women would have over 90% power (two-sided alpha = 5%) to detect a clinically important 25% reduction from 10 to 7.5% in PPH. The sample size estimate is based on two key assumptions (1) the baseline event rate and (2) the size of the treatment effect. The primary endpoint is PPH. The prevalence of PPH is estimated at 6% world-wide but 10% in Africa and Asia [2]. If the event rate is 10% then the trial has 99% power. However, if the event rate is lower, the study will have less power. For example if the 6% estimate applies, the trial would have just over 90% power. Planning for the possibility that the event rate may be lower than anticipated is a sensible precaution. It is also possible that the treatment effect is not as large as predicted. Although a 25% reduction would be clinically important, a more modest reduction would also be worthwhile. The additional power also reduces the chance that a more modest treatment effect will be missed. Experience from the WOMAN trial shows that loss to follow-up will be minimal (less than 1%) and will not influence trial power. The LSHTM Clinical Trials Unit (CTU) has successfully recruited large sample sizes for previous international trials and is experienced at managing recruitment at sites to ensure that target recruitment is achieved. The main anticipated risk to recruitment to the WOMAN-2 trial is political instability in the participating countries. The main resource for mitigating this risk is the large international network of obstetric clinical trialists that was established during the WOMAN trial. If political instability prevents the recruitment of patients in any of the planned settings, recruitment in other sites will be initiated, thereby reducing the risk.

### Identification of participating investigators and trial sites

Participating investigators and trial sites will be identified from the international network of obstetricians that was established during the WOMAN trial and includes hospitals where anaemia in pregnancy is common. Before the trial can start at any site, all relevant regulatory and ethics approvals must be in place and the site principal investigator (PI) must agree to conduct the trial according to the Protocol, Good Clinical Practice (GCP) guidelines and all the relevant regulations. Names of participating sites will be listed on the trial website (http://woman2.lshtm.ac.uk/).

### Eligibility of participants

#### Inclusion criteria

Women with moderate or severe anaemia (Hb level < 100 g/L or packed cell volume (PCV) < 30%), who have given birth vaginally and for who the responsible clinician is substantially uncertain whether to use TXA.

#### Exclusion criteria

- Women who are not legally adult (< 18 years) and permission not provided by a guardian
- Women with a known allergy to TXA or its excipients
- Women who develop PPH before the umbilical cord is clamped/cut

### Information giving and consent procedure

Wall posters and brief information leaflets (Additional file 3) will be used to inform pregnant women attending antenatal clinics and labour wards about the trial. Information may also be provided in videos. Eligible women with moderate or severe anaemia will be invited to take part in the trial. If women are in the active stage of labour and able to give fully informed consent, written consent will be obtained. However, many women arrive at hospital in the second stage of labour. Because these women are more likely to be anaemic and more likely to have a PPH it will be important to include these women in the trial. However, they may not have the physical or mental capacity to give fully informed consent due to the pain of labour, poor health or the urgency of the situation. In these cases, a clinician will assess the capacity of the woman and the most appropriate consent procedure will be used for her. An overview of the consent procedure is provided in Additional file 4.

The consent procedure will be adapted depending on the clinical situation and the capacity of each woman. The consent process used will be documented in the participant’s medical records.

#### Potentially eligible woman identified in active labour

A member of the site trial team will approach the woman with the agreement of the primary carer. She will be given information about the trial (Additional file 5) and the trial will be discussed in a language she understands. The team member will explain the purpose of the trial, that it does not involve any change to her birth plan, and that she will receive all the usual interventions for preventing PPH and any other care she needs. The team member will explain that her participation is voluntary and that if she does not want to take part that we will respect her views and that her decision will not affect her care. If she wants to take part, the team member will obtain written consent. If she is unable to read or write, the information sheet will be read to her and she will mark the consent form with a cross or thumbprint. In this case, an impartial witness must provide a signature confirming the mark. A copy of the information sheet and consent form will be given to the woman. If a woman is in active labour and her capacity to consent is impaired, for example by analgesia or pain, consent will be obtained as described in the following section.

#### Potentially eligible woman identified in the second stage of labour

Many women in LMICs arrive at hospital in the second stage of labour. A study in Nigeria found that over one quarter of women arrived at the labour ward in the second stage [24]. Furthermore, women who arrived in the second stage were more often anaemic and at greater risk of PPH. However, many of these women will be unable to give fully informed consent due to pain, medication or the urgency of the situation. Fully informed consent will be obtained if her physical and mental capacity allows. If in the view of the responsible clinician a woman is unable to give fully informed consent, information to her level of capacity will be given and her verbal agreement obtained in the presence of an impartial witness. If an accompanying person (e.g. her partner or other family members) is present, their views will be considered but the opinion of the woman will prevail. Where verbal agreement has been obtained, a woman can be enrolled into the study. Fully informed consent for ongoing trial participation will be sought from the woman as soon as possible after she regains her full capacity.

#### Women under 18 years old

If a woman is under 18 years of age, her consent will be witnessed by a guardian who should be an appropriate responsible adult (e.g. her parents, husband, partner or other family member) who will countersign the consent form.

#### Withdrawal of consent

If the woman withdraws a previously given informed consent, refuses to consent for continuation in the trial, or if the woman dies and no written consent is available, her data will be handled as follows:

- Data collected to the point of withdrawal of consent will be used as part of the intention to treat analysis
- In the event a woman dies and full consent is not available, data will be collected as per protocol as the woman would have given her agreement to take part in the trial. The relevant Ethics Committee will be informed

### Screening and enrolment procedures

#### Routine clinical screening

Many pregnant women are likely to arrive to give birth at a participating hospital without antenatal care, or with low compliance with treatments for anaemia. It is important for her clinical care that her Hb or PCV value is known before giving birth. If no test has been done on admission to give birth, women planning to give birth vaginally will be offered a standard point-of-care Hb assessment (HemaCue®). Pregnant women will be informed about the purpose of the test before it is performed and they will have the right to accept or decline in line with any clinical care being offered. The test will be provided free of charge. Information on patients screened will be recorded on a Screening Log.

Women with a moderate or severe anaemia (Hb level < 100 g/L or PCV < 30%) will be offered the opportunity to participate in the WOMAN-2 trial.

#### Baseline screening and eligibility confirmation

Following completion of the appropriate informed consent procedure, data on demographics, anthropometry, clinical signs, pregnancy and medical history, risk factors for postpartum haemorrhage, about the birth, about the baby/ies and baseline treatment plan for the anaemia, will be collected in the Case Report Form (CRF) Booklet. Some data will be collected before a woman gives birth which will assess potential eligibility. Final eligibility will be confirmed at delivery of the baby’s anterior shoulder up to when the cord is clamped or cut. This is because some women who plan to deliver vaginally and have provided consent may need a caesarean section or may develop PPH before the cord is cut or clamped which will make them ineligible for the trial.

### Randomisation

An IT coding expert supported by a statistician who are not involved in the conduct of the trial will prepare the randomisation codes. They will give a copy to the sponsor’s representative, who is also not associated with the conduct of the trial, for manual back-up. The IT coding expert will also send the codes to the trial drug manufacturer so that treatment packs can be prepared in accordance with the randomisation list. Trial staff (coordinating centres and sites) and patients will not have access to the randomisation codes until final database lock or unless un-blinding of an individual patient is requested.

Women who are eligible for inclusion will be randomised to receive active (TXA) or placebo (sodium chloride 0.9%) by intravenous injection.

Once eligibility has been confirmed at delivery of the baby’s anterior shoulder and up to when the cord is clamped/cut, the next lowest consecutively numbered pack will be taken from a box of 20 treatment packs. The participant is considered randomised to the trial once administration of the trial treatment has started. Each site will keep a log of women they randomise to the trial. Site investigators will need to explain any out-of-sequence use of the trial treatment.

### Trial treatment

#### Name and description of Investigational Medicinal Product (IMP)

Tranexamic acid (TXA) is a synthetic derivative of the amino acid lysine that exerts an antifibrinolytic effect through the reversible blockade of lysine binding sites on plasminogen molecules. TXA is sold under a variety of trade names for the treatment of bleeding due to general or local fibrinolysis in adults and children from 1 year of age [25]. TXA would be given in addition to all the usual interventions for preventing PPH, thus WOMAN-2 will compare its effects with matching placebo (sodium chloride 0.9%) to ensure blinding.

#### Drug administration and dosage schedule

A single dose of 1 g of TXA or placebo (sodium chloride 0.9%) by intravenous injection will be given immediately after the umbilical cord is cut or clamped, and no more than 15 min later. There should be no delay in administering the trial medication after the umbilical cord is cut or clamped. Each treatment pack contains two ampoules each containing 500 mg (5 mL) of TXA or placebo (5 mL), and one sterile 10-mL syringe and 21-G needle. Appropriately qualified staff will prepare the treatment to be administered by drawing up the contents of both ampoules into the 10-mL syringe using the 21-G needle provided. Before administration, the expiry date will be checked and the randomisation number confirmed. The contents of both ampoules (total volume 10 mL) will be administered as a slow intravenous injection at rate of about 1 mL/min using a standard local intravenous administration procedure.

In the event of multiple births, the trial drug will be given after cutting or clamping the umbilical cord of the last baby.

#### Known drug reactions and interaction with other therapies

TXA should not be mixed with other medicinal products.

#### Trial restrictions and the use of concomitant medication

Women should receive all clinically indicated treatments. There is no restriction on the use of concomitant medication. Many women will develop PPH and these women should be treated in the usual way, which may include TXA. If any contra-indication to the trial treatment develops after randomisation, the trial treatment should be stopped.

#### Assessment of compliance

Trial team members at each site will record the date and time of trial treatment administration. If the trial treatment is not given, or is given outside of the prescribed time period, a reason will be required.

#### Risks and benefits

TXA reduces the risk of death due to bleeding in women with PPH. The WOMAN trial randomised 20,060 women with PPH to receive TXA or placebo [26]. The results show that TXA significantly reduces death due to bleeding (RR = 0.81, 95% CI 0.65 to 1.00), particularly when given within 3 h of giving birth (RR = 0.69, 95% CI 0.52 to 0.91). There is also evidence from randomised trials that TXA improves outcomes in traumatic and surgical bleeding. The CRASH-2 trial of TXA in 20,211 bleeding trauma patients showed that TXA reduces death due to bleeding when given soon after injury [27, 28]. Treatment within 3 h of injury reduced the risk of death due to bleeding by around 30% (RR = 0.72, 95% CI 0.63 to 0.83). In surgery, a systematic review of 129 randomised trials found that TXA reduces the probability of receiving a blood transfusion by 38% (RR = 0.62, 95% CI 0.58 to 0.68) and average blood loss by 34% (RR = 0.66, 95% CI 0.65 to 0.67) [15, 29]. There was no increased risk in AEs with TXA in either the WOMAN trial, the CRASH-2 trial or the surgical systematic review.

TXA is widely used and well tolerated. Potential side-effects reported by manufacturers to be associated with use of TXA according to frequency [30] are

- Common (≥ 1/100 to < 1/10): diarrhoea, vomiting and nausea
- Uncommon (≥ 1/1000 to < 1/100): allergic dermatitis
- Rare: hypersensitivity reactions including anaphylaxis; convulsions; visual disturbances including impaired colour vision; malaise with hypotension (generally following an intravenous injection given too rapidly); arterial or venous thrombosis

There is some evidence from cohort studies in cardiac surgery patients that high doses of TXA are associated with seizures [31–33]. The doses used in these studies (6–20 g) were substantially higher than the dose that will be used in the WOMAN-2 trial (1 g). There was no increased risk of seizures with TXA in the two large trials involving over 40,000 participants that used 1–2 g of TXA (WOMAN and CRASH-2 trials).

Women in the postpartum period have an increased risk of thromboembolic events compared with non-pregnant women. Although the absolute risk of venous thrombosis is low at around 2 per 1000 woman-years, women in the postpartum period are four times more likely to suffer a venous thrombosis than non-pregnant women of the same age [34]. Although on mechanistic grounds TXA has the potential to increase the risk of venous thrombosis, randomised trials provide no evidence of any increased risk of venous thrombosis with TXA. In the WOMAN trial, the risk of venous thrombosis did not differ significantly between groups (Table 1). Because severe bleeding is a strong risk factor for vascular occlusive events and TXA reduces bleeding, it is possible that TXA reduces (rather than increases) the risk of thrombosis [35].

**Table 1:** Effect of tranexamic acid (TXA) on thromboembolic events in the WOMAN trial [13]

	TXA (*n* = 10,033)	Placebo (*n* = 9985)	RR (95% CI)	*P* value
Any thromboembolic event	30 (0.3%)	34 (0.3%)	0.88 (0.54 to 1.43)	0.603
Venous events	20 (0.2%)	25 (0.3%)	0.80 (0.44 to 1.43)	0.446
DVT	3 (0.03%)	7 (0.07%)	0.43 (0.11 to 1.65)	0.203
PE	17 (0.2%)	20 (0.2%)	0.85 (0.44 to 1.61)	0.611
Arterial events	10 (0.1%)	9 (0.09%)	1.11 (0.45 to 2.72)	0.827
Myocardial infarction	2 (0.02%)	3 (0.03%)	0.66 (0.11 to 3.97)	0.651
Stroke	8 (0.08%)	6 (0.06%)	1.33 (0.46 to 3.82)	0.599

*CI* confidence interval, *DVT* deep vein thrombosis, *PE* pulmonary embolism, *RR* Relative Risk

TXA is excreted in the urine unchanged with 90% of the dose excreted in the 12 h after administration. Plasma concentrations are higher in renal insufficiency and with repeated dosing there is a risk of accumulation. However, because a single dose of 1 g is used in the WOMAN-2 trial, there will be no risk of accumulation. Unpublished data from the manufacturer indicate that TXA passes into breast milk at a concentration of approximately 100th of the concentration in the maternal blood and thus an antifibrinolytic effect in the infant is unlikely [21, 22]. There was no increase in adverse effects in infants of mothers who received TXA in the WOMAN trial. Observational studies of TXA use during breastfeeding also found no adverse effects [36].

#### Investigator’s Brochure (IB)

Information about TXA and the manufacture of the placebo will be detailed in an Investigator’s Brochure (IB). The IB will be reviewed annually. Any studies that provide reliable information on the safety and efficacy of TXA that would help investigators to assess the risks and benefits of TXA use, will be included. Additionally, information on updates from relevant manufacturers of TXA will be included. Information relevant to the safety and wellbeing of the participants or the scientific value of the trial will be communicated to investigators, Data Monitoring Committee (DMC), Ethics Committees and regulatory authorities.

#### Preparation and labelling of medication to be used in the trial

TXA which has Marketing Authorisation in the United Kingdom (UK) will be purchased from the open market. Marketing Authorisation guarantees that drug manufacture and release complies with Good Manufacturing Practice (GMP). A GMP-certified manufacturer will prepare the matching placebo (sodium chloride 0.9%). Ampoules and packaging will be identical in appearance for both TXA and placebo. A clinical trial supply company will carry out the blinding process and first-stage Qualified Person (QP) release. The blinding process involves replacing the manufacturer’s label with the clinical trial label that has the randomisation number (used as pack identification). Other label text will be identical for TXA and placebo and complies with requirements for clinical trials. Checks on a random sample of drug packs to check the blinding will be carried out to compare known TXA with blinded samples to confirm which ones are TXA. The samples will then be un-blinded to assure accuracy of the labelling.

#### Drug storage and supply

When a site is ready to start, a box containing 20 trial treatment packs will be sent by the CTU. Thereafter, site stock level will depend on the site’s average recruitment rate. Each time a participant is randomised and the randomisation data are entered in the trial database, one pack from the site’s stock will be automatically deducted. When stock reaches the site’s minimum level, the CTU will send another box (or boxes). Sites will need to send screening and entry data to the CTU as soon as possible after randomisation (ideally within 24 h). Sites will have to report all used trial treatments packs and those that are lost or damaged, to the CTU on a Drug Accountability Log (DAL).

At each site, the trial treatment packs will be stored securely, but in a place where they are accessible to the trial team for randomisation at all times. Although TXA is heat stable, it will be stored in a dry place where it is protected from excessive heat and freezing.

The expiry date of the trial treatment pack will be printed on the ampoule label, the treatment pack and the drug box. When a batch of treatment packs is close to expiry, the PI/trial pharmacist/delegate will be asked to arrange destruction of affected packs and record this. When a site is to be closed, the PI/trial pharmacist/delegate will arrange destruction of all unused packs and confirm disposal to the CTU.

### Un-blinding

In general, there should be no need to un-blind the allocated intervention. Un-blinding should occur only in those rare cases when the clinician believes that a participant’s management depends importantly upon knowledge of whether the participant received TXA or placebo. If a woman in the trial develops PPH, she should receive all clinically indicated treatments. There should be no need to un-blind before initiating treatment with TXA. Even if a woman received 1 g of TXA immediately after giving birth, TXA has a large therapeutic index and a second 1-g dose is well within the dosing range. In those few cases when urgent un-blinding is necessary, a 24-h telephone service is provided by the CTU. The caller will receive a voice message, text message or email informing them whether the woman received TXA or placebo. The investigator should complete an un-blinding request/report form within five working days of un-blinding. If a suspected unexpected serious adverse reaction (SUSAR) is reported, un-blinding may be needed for reporting to Regulatory Agencies and Ethics Committees.

### Outcome measures

Once randomised, we will collect follow-up data even if the trial treatment is not completed. Data will be collected within the first 24 h after administration of the trial treatment and final outcome data will be collected when a woman is discharged from the randomising hospital, at death or 42 days post randomisation, whichever occurs first. In the event a woman is discharged or dies within 24 h, all outcomes will be assessed at the same time. Adverse events will be collected from administration of the trial medication up to day 42.

#### Primary outcome

The primary outcome is a clinical diagnosis of primary PPH. This may be an estimated blood loss of more than 500 mL or any blood loss sufficient to compromise haemodynamic stability within 24 h of administration of trial medication. Haemodynamic instability is based on clinical judgement and assessed using clinical signs (low systolic blood pressure, tachycardia, reduced urine output). The cause of PPH will be described.

#### Secondary outcomes

Maternal blood loss and its consequences:

- Postpartum blood loss (clinical estimation)
- Haemoglobin
- Haemodynamic instability
- Shock index
- Receipt of blood transfusion
- Use of interventions to control postpartum bleeding (medical and surgical)

Maternal health and wellbeing:

- Symptoms of anaemia (e.g. fatigue, headache, dizziness, palpitations, breathlessness)
- Exercise tolerance (short 6-min walk test)
- Quality of life (overall wellbeing, ability to care for herself and her baby, breastfeeding)

Other health outcomes:

- Vascular occlusive events (pulmonary embolism (PE), deep vein thrombosis (DVT), stroke, myocardial infarction (MI))
- Organ dysfunction
- Sepsis
- Expected side effects (nausea, vomiting, diarrhoea, seizure)
- Adverse events
- Death (cause and time to death will be described)
- Length of hospital stay
- Admission to and time spent in higher level facility
- Status of baby/ies and any thromboembolic events

### Data management

#### Source data

Source documents include, but are not limited to, hospital records (from which medical history, previous and concurrent medication, clinical outcomes and AEs may be summarised onto the CRFs), clinical and office log books, laboratory and pharmacy records, diaries and correspondence. CRF entries will be considered source data if the CRF is the site of the original recording (e.g. quality of life questionnaire, 6-min walk test, breathlessness score). Trial data will be kept confidential and stored securely. On all trial-specific documents, other than the consent form, the participant will be referred to by the trial participant screening ID number and not by name.

#### Access to source data

Direct access will be granted by participating sites to authorised representatives from the sponsor, host institution and the regulatory authorities to permit trial-related monitoring, audits and inspections.

#### Data recording and record keeping

All trial data will be entered on to paper CRFs and then entered onto the trial database by authorised site staff. The CRFs can be viewed at http://woman2.lshtm.ac.uk/. The participants will be identified by a unique trial-specific number. The name and any other identifying detail will not be included in trial data electronic file used for analysis or publication. An Investigator’s Site File (ISF) containing the essential documents for the trial will be provided by the CTU. The ISF must be updated by the trial site throughout the course of the trial.

#### Participant confidentiality

The trial staff will ensure that participants’ confidentiality is maintained. Participants will be identified only by a participant screening ID number on all trial documents and any electronic database, with the exception of the paper CRF which remains at participating sites, where participant initials may be added. All documents will be stored securely and only accessible by trial staff and authorised personnel. The trial will comply with relevant data protection regulations including the UK General Data Protection Regulation.

#### Serious breaches and protocol deviations

A serious breach is defined as ‘a breach of GCP or the trial protocol which is likely to affect to a significant degree (a) the safety or physical or mental integrity of the subjects of the trial; or (b) the scientific value of the trial’.

In the event that a serious breach is suspected, the site must inform the CTU within one working day. The CTU will report all serious breaches to the relevant RECs, regulatory authorities within the timeline required by each participating country.

A protocol deviation is a departure from the approved protocol’s procedures made with or without prior approval. Such departures may be major or minor/administrative in nature. All deviations must be reported to the CTU within 24 h of it becoming known to the trial team.

### Follow-up and outcome assessment

Trial follow-up ends at hospital discharge, death or 42 days post randomisation, whichever comes first. However, AE reporting will continue up to day 42. Date and time of assessments will be recorded.

#### Primary outcome

The primary outcome will be a clinical diagnosis of PPH within 24 h of administration of the trial treatment or at discharge from hospital, whichever is earlier. This may be an estimated blood loss of more than 500 mL or any blood loss sufficient to compromise haemodynamic stability within 24 h of delivery. Haemodynamic instability is based on clinical judgement and assessed using clinical signs (e.g. low systolic blood pressure, tachycardia, reduced urine output). The cause of PPH will be described.

#### Secondary outcomes

The following secondary outcomes will be assessed at 24 h after administration of trial treatment or discharge from hospital, whichever is earlier:

- Postpartum blood loss: clinical estimation of blood loss since administration of trial treatment
- Haemoglobin: using HemaCue® point-of-care test
- Haemodynamic instability (within 24 h of administration of trial medication): presence of haemodynamic instability based on clinical signs, e.g. low blood pressure, tachycardia, reduced urine output requiring intervention (e.g. intravenously administered fluid)
- Shock index – heart rate/systolic blood pressure: The lowest recorded systolic blood pressure and the corresponding heart rate

The following outcomes will be assessed at death, discharge from hospital or 42 days, whichever is earlier:

- Quality of life: the following parameters will be measured by questionnaire, overall wellbeing, ability to care for herself and her baby, and breastfeeding
- Symptoms of anaemia: the following parameters will be assessed by questionnaire, fatigue, headache, dizziness, palpitations, breathlessness, flaring of the alae nasi
- Expected side effects of trial medication: the following side effects will be recorded, nausea, vomiting, diarrhoea
- Exercise tolerance: assessed using the 6-min walk test [37]
- Blood transfusion: number of units given (units started before administration of trial medication will not be included). Information on type of transfusion will be collected.
- Use of interventions to control primary postpartum haemorrhage (medical and surgical): including uterotonics, removal of placenta/placental fragments, intrauterine balloon tamponade, bimanual uterine compression, external aortic compression, non-pneumatic anti-shock garments, uterine artery embolisation, uterine compression suture, hysterectomy and laparotomy to control bleeding
- Vascular occlusive events: including pulmonary embolism (PE), deep vein thrombosis (DVT), stroke and myocardial infarction (MI):
  - ○ PE: diagnosis will be confirmed by radiological examination
  - ○ DVT: diagnosis will be confirmed by ultrasound or radiological examination
  - ○ Stroke: defined as ‘a new focal neurological deficit with signs and symptoms lasting more than 24 h’
  - ○ MI: diagnosis in the presence of one of the following: (1) electrocardiogram (ECG) showing unequivocal pathological Q waves and/or ST-segment elevation or depression in serial recordings; (2) history of typical or atypical angina pectoris, together with equivocal changes on the ECG and elevated enzymes; (3) history of typical angina pectoris and elevated enzymes with no changes on the ECG or not available; (4) fatal cases, whether sudden or not, with naked-eye appearances of fresh MI and/or recent coronary occlusion at necropsy (antemortem thrombus, haemorrhage into an atheromatous plaque or embolism)
- Organ dysfunction:
  - ○ Cardiovascular dysfunction – shock, cardiac arrest (absence of pulse/ heart beat and loss of consciousness), use of continuous vasoactive drugs, cardiopulmonary resuscitation, severe hypoperfusion (lactate > 5 mmol/L or > 45 mg/dL), severe acidosis (pH < 7.1)
  - ○ Respiratory dysfunction – acute cyanosis, gasping, severe tachypnea (respiratory rate > 40 breaths per min), severe bradypnoea (respiratory rate < six breaths per min), intubation and ventilation not related to anaesthesia, severe hypoxemia (O_2_ saturation < 90% for ≥ 60 min or PAO_2_/FiO_2_ < 200)
  - ○ Renal dysfunction – oliguria non-responsive to fluids or diuretics, dialysis for acute renal failure, severe acute azotaemia (creatinine ≥ 300 μmol/mL or ≥ 3.5 mg/dL)
  - ○ Coagulation/haematological dysfunction – failure to form clots, massive transfusion of blood or red cells (≥ 5 units), severe acute thrombocytopenia (< 50,000 platelets/mL)
  - ○ Hepatic dysfunction – jaundice in the presence of eclampsia, severe acute hyper-bilirubinaemia (bilirubin > 100 μmol/L or > 6.0 mg/dL)
  - ○ Neurological dysfunction – prolonged unconsciousness (lasting ≥ 12 h)/coma (including metabolic coma), stroke, uncontrollable fits/status epilepticus, total paralysis
- Sepsis: diagnosis is based on the presence of both infection and a systemic inflammatory response syndrome (SIRS). SIRS requires two or more of the following: (1) temperature < 36 °C or > 38 °C, (2) heart rate > 90 beats/min, (3) respiratory rate > 20 breaths/min, (4) white blood cell count < 4 × 10^9^/L (< 4000/mm^3^) or > 12 × 10^9^/L (> 12,000/mm^3^)
- In-hospital death: cause and time of death will be described
- Length of hospital stay
- Admission to, and time spent in, a higher-level facility: higher-level facilities include High-dependency and Intensive Care Units
- Status of baby/ies: the status (dead/alive)
- Any thromboembolic events in breastfed babies (may include any venous or arterial thrombosis (thrombosis of limb artery/deep veins, renal artery/veins, pulmonary embolism, hepatic veins, caval veins, intracardiac thrombosis, portal vein, mesenteric veins/artery, cerebral veins, retinal vein, ischemic stroke, arteries, aorta, myocardial infarction, microvascular thrombosis from purpura fulminans or disseminated intravascular coagulation)
- Adverse events

### Safety reporting

Outcome events recorded on the CRF Booklet up to death, discharge or day 42 (whichever is sooner) will not be included in the definitions of AEs detailed below. These outcome events include PPH, anaemia, vascular occlusive events, organ dysfunction, sepsis, nausea, vomiting, diarrhea and seizure.

The CTU will present data on these outcome events to the independent DMC for regular review. These outcome events will not be reported using the AEs’ reporting procedure. However, all other medical events fulfilling the AE definition will be reported up to 42 days after administration of trial treatment. In the event that a woman is discharged before 42 days, AEs to be reported after discharge will also include all outcome events.

At discharge, participants will be given an ‘alert card’ that identifies them as a WOMAN-2 participant, and asked to present this card to anyone providing medical care after discharge, up to day 42. Instructions to ensure the AEs’ reporting procedures for the trial are followed will be detailed on the card. An overview of the safety reporting procedure is presented in Additional file 6.

#### Definitions

##### Adverse event (AE)

Any untoward medical occurrence in a participant to whom a medicinal product has been administered including occurrences which are not necessarily caused by or related to that product. An AE can, therefore, be any unfavourable and unintended sign (including an abnormal laboratory finding), symptom, or disease temporally associated with the use of an IMP,.

##### Adverse reaction (AR)

Any untoward and unintended response in a participant to an IMP which is related to any dose administered to that participant.

The phrase ‘response to an investigational medicinal product’ means that a causal relationship between a trial medication and an AE is at least a reasonable possibility, i.e. the relationship cannot be ruled out.

##### Serious adverse event (SAE)

A SAE is any untoward medical occurrence that results in death; is life-threatening; requires inpatient hospitalisation or prolongation of existing hospitalisation; results in persistent or significant disability/incapacity other ‘important medical events’ may also be considered serious if they jeopardise the participant or require an intervention to prevent one of the above consequences.

##### Serious adverse reaction (SAR)

An AE that is both serious and, in the opinion of the reporting investigator, believed with reasonable probability to be due to the trial treatments, based on the information provided.

Suspected unexpected serious adverse reaction (SUSAR) – a serious adverse reaction, the nature and severity of which is not consistent with the information about the medicinal product in question set out in the in the Investigator’s Brochure (IB).

#### Causality

When completing the AE reporting form, the site PI or medical delegate will assign a causality using the definitions below:

- Suspected to be related – There is evidence to suggest a causal relationship with administration of the trial treatment and the influence of other factors is unlikely
- Not suspected to be related – There is little or no evidence to suggest there is a causal relationship (e.g. the event did not occur within a reasonable time after administration of the trial treatment). There is another reasonable explanation for the event (e.g. the participant’s clinical condition, other concomitant treatment)

If there is any doubt about the causality, the site PI or medical delegate will inform the CTU. In the case of discrepant views on causality between the investigator and others, all parties will discuss the case. In the event that no agreement is made, both points of view are to be recorded and reported onwards as required.

#### Reporting procedures

##### Adverse reactions (ARs)/adverse events (AEs)

Adverse Event Reporting Forms will be provided in the CRF Booklet. Site investigators will report non-serious ARs and AEs using these.

##### Serious adverse reactions (SARs)/serious adverse events (SAEs)

Adverse events and ARs which fulfil the serious criteria will be reported to the CTU within 24 h of the PI or delegate becoming aware of the event using the Adverse Event Reporting Form. The form will be completed and submitted to the CTU with as much detail of the event that is available at that time. If awaiting further details, a follow-up report will be submitted promptly upon receipt of any additional information (but no later than five working days of becoming aware of the event). The site PI or medical delegate must record the event with an assessment of seriousness, causality and expectedness. Events relating to a pre-existing condition or any planned hospitalisations for elective treatment of a pre-existing condition will not be reported as SAEs.

##### Suspected unexpected serious adverse reactions (SUSARs)

All SAEs assigned by the site PI or medical delegate as suspected to be related to the trial treatment and which are unexpected, will be classified as SUSARs and will be subject to expedited reporting to each participating regulatory authority, Ethics Committees and the sponsor within seven working days of being reported to the CTU.

In the case of a SUSAR, the staff at the site will:

- Contact the CTU immediately by telephone or email to inform them of the event and obtain guidance on the reporting procedure if needed
- Submit an Adverse Event Report, completed with all available information (signed and dated) within 24 h, together with relevant treatment forms and anonymised copies of all relevant clinical investigations
- Submit any additional information promptly upon request
- Emergency contact details for advice on reporting SAEs and SUSARs can be found in the Investigator’s Study File

Adverse Event Reporting Forms are submitted in the following ways:

- Directly via the trial database (see Investigator’s Study File for full details)
- Fax: + 44 (0)20 7299 4663
- Email: woman2.data@lshtm.ac.uk

AEs considered related to the trial medication as judged by a site investigator or the CTU will be followed either until resolution, or the event is considered stable.

#### Adverse event reporting to the relevant authorities

All SUSARs will be reported by the CTU or sponsor representative to the relevant regulatory authority and REC and other parties as applicable. For fatal and life-threatening SUSARS, this will be done no later than seven calendar days after the CTU is first made aware of the reaction. Any additional relevant information will be reported within eight calendar days of the initial report. All other SUSARs will be reported within 15 calendar days. Treatment codes will be un-blinded for specific participants if required. Site PIs will be informed of all SUSARs for all studies using TXA sponsored by the LSHTM, whether or not the event occurred in the WOMAN-2 trial.

All other AEs will be reported as requested by the relevant authorities.

### Withdrawal criteria

The trial ends for a participant at death or at day 42 whichever occurs first. A participant can leave the trial at any time. A participant may provide the research team with the reason(s) for leaving the study, but is not required to do so. The trial team will inform the participant to return if she has any medical concerns. If a participant decides to withdraw from the trial, data collected up to the point of withdrawal will be used as part of the intention-to-treat analysis, but no other data will be collected unless the woman gives her permission to do so. In all cases, her wishes will be respected.

### Definition of end of trial

The end of trial will be day 42 of the last participant randomised.

### Monitoring

#### Risk assessment

Data on side effects that might be associated with anaemia, postpartum bleeding or TXA use will be collected and will be presented to the independent DMC. The trial involves seeking consent, giving the trial drug using a routine clinical procedure, collecting outcome information (mostly from the hospital notes), a Hb estimation, timed walk test, and a quality of life questionnaire. Prior to discharge, the woman’s Hb concentration will be measured using a point-of-care test (HemaCue®). This involves a pinprick blood sample and takes only a few minutes. This test will provide clinically useful information for the patient and treating physicians.

The timed walk test assesses the woman’s functional exercise capacity. The test measures the distance that a woman can walk on a flat, hard surface in a period of 6 min. A chair will be placed along the walking area so she can rest during the test if she chooses to. The results should give a good indication of the woman’s ability to carry out her daily physical activities. The test is safe in patients with cardiopulmonary disease and in frail older populations. The test will not be done if there is a history of unstable angina or myocardial infarction in the past month but these conditions will be extremely rare in postpartum women. Apart from the trial drug, all other clinical care will be as per usual practice. For these reasons, the risk of harm or injury (whether physical, psychological, social or economic) from participating in the trial is assessed to be low.

The outcome questionnaire will ask participants about their mental and physical wellbeing, and breastfeeding. It is possible that these questions may bring up feelings for participants such as discomfort, worry or sadness. Trial staff will be trained to ask the questions in a sensitive manner and to look for signs of discomfort in participants. If the trial team member detects discomfort in any participant, they will ask permission to continue. If the trial team member has any concerns about the wellbeing of the participant, they should refer her back to the treating clinician for further assessment and ongoing care.

#### Central monitoring

The trial will be conducted in accordance with the current approved protocol, GCP, relevant regulations guidance provided in the Investigator’s Study File and the trial’s standard operating procedures.

A detailed monitoring plan will be developed. In summary, the CTU will closely monitor the trial to ensure the rights, safety, and wellbeing of the trial participants and to ensure the accuracy of the data. All coordinating centres and site trial teams will be trained in the trial procedures and provide extensive guidance. Central monitoring methods will be used by the CTU. A sample of consent forms from all sites will be monitored at the CTU to make sure they are properly completed. In addition, data management and statistical checks of data (central statistical monitoring) will be done to ensure that trial participants meet the inclusion criteria and trial treatment is administered in line with the protocol. Event rates for primary and secondary outcomes will be monitored. Sites with higher or lower than expected event rates will be selected for further monitoring. Quantitative variables (systolic blood pressure (SBP), heart rate (HR), respiratory rate and blood loss) will be monitored to check the accuracy of the data. For example, the coefficient of variation for the data at each site will be examined and those where there is any reason for concern will be selected for further monitoring.

#### Monitoring at local site

Onsite monitoring will be carried out at any site flagged as high risk on central statistical monitoring and other central monitoring procedures. Source data verification will be done on at least 10% of the trial data. Additionally, site self-monitoring will be carried out where needed. This will involve the PI/delegate at a site monitoring themselves against a standardised checklist. The LSHTM CTU will require investigators and their institutions to provide access to source data and all trial-related documents for monitoring, audits, Ethics Committee review and regulatory inspection. All trial-related and source documents including medical records, original consent forms and original CRFs must be kept safely. Investigators must plan in advance of the trial start where the trial-related documents will be stored and how they will be accessed. All documents must be made available when required for monitoring/audit/inspection during the course of the trial and for up for 5 years after the end of the overall trial.

### Trial closure

Trial closure will happen in the following circumstances:

- Routine – in preparation for the completion of a trial
- Unscheduled – as a result of failure to obtain continuation funding, DMC request and Trial Steering Committee agreeing based on negative or positive findings, findings in other studies that impact on this trial, or other unforeseen events (e.g. safety concerns, civil unrest, etc.)

### Statistics and data analysis

A detailed Statistical Analysis Plan (SAP) will be drafted and agreed with the DMC for their ongoing review and will be finalised before the trial database is locked for final analysis.

#### Main analysis

Analyses will be on an ‘intention-to-treat’ basis. Data will be analysed by randomised group, irrespective of whether the group received the intervention. Demographic and other baseline characteristics will be tabulated. Descriptive statistics for continuous variables will include the mean, standard deviation, median, range, and number of observations. Categorical variables will be presented as numbers, and as percentages of those participants who had the assessment. All statistics will be presented by treatment group. Effect measures will be Relative Risk (RR) and absolute risk reduction. Precision will be quantified using 95% confidence intervals. Planned subgroup analyses include analyses based on the severity of anaemia (moderate versus severe) and type of labour (induced or augmented versus spontaneous). In a large trial, such as WOMAN-2, the baseline characteristics of participants that may influence the outcome are expected to be evenly distributed between the treatment and placebo groups, so that any difference in outcome can be attributed to the intervention. However, it is still possible that a chance imbalance in important prognostic factors could influence the results. To investigate this possibility, an analysis of the effect of treatment that is adjusted for baseline risk will be conducted. A prognostic model will be built based on pre-specified baseline variables and use it to estimate the predicted risk of the outcome at baseline. Checks will be made to ensure that there are sufficient patients in the severe anaemia subgroup by limiting recruitment to these patients if necessary. For subgroups, RRs and confidence intervals with two-sided *P* values will be reported. Test of homogeneity of effect across the subgroups will be done and a *P* value reported. Unless there is evidence against the null hypothesis of homogeneity of effects the overall RR will be taken as the most reliable guide to the approximate RR in all subgroups.

### Regulatory issues

#### Approvals

Approval from the LSHTM Research Ethics Committee (REC) has been obtained. Approval will be obtained from the relevant RECs and regulatory authority of each participating country. Where site approval is needed, this will be obtained before the trial starts.

#### Confidentiality

Any identifiable data obtained by the CTU will be stored securely and confidentiality protected in accordance with the UK General Data Protection Regulation 2018. Local investigators will collect consent, baseline, outcome and adverse event data and will send them to the CTU. Investigators will transmit data to the CTU by entering them into the online trial database. Investigators will be given a unique username, password and PIN to access the database. The CTU will securely store copies of consent forms sent for monitoring and these will be destroyed at trial closure. Original copies of CRFs, consent forms and source data will be kept securely at each participating site. These must be archived securely for 5 years after the overall end of the trial. Only people authorised by the project director/project manager will have access to the WOMAN-2 trial database. The trial database will be accessed through a complex password system which includes a password ageing mechanism (i.e. passwords will be changed every 90 days).

#### Indemnity

The LSHTM accepts responsibility attached to its sponsorship of the trial and, as such, would be responsible for claims for any non-negligent harm suffered by anyone as a result of participating in this trial. The indemnity is renewed on an annual basis and the LSHTM assures that it will continue renewal of the indemnity for the duration of this trial.

#### Sponsor

The London School of Hygiene and Tropical Medicine (LSHTM) will act as the sponsor for this trial. Contact details are provided in Additional file 7.

#### Funding

Wellcome (WT208870/Z/17/Z) and the Bill & Melinda Gates Foundation (OPP1176150) are funding this study. Women will not be paid for taking part as there is no special travelling or time off work needed. A small practical gift as a ‘thank you’ will be given to women who participate. The monetary value of the gift will not exceed three British pounds. Where a woman returns to hospital for any adverse event associated with the trial, her travel costs will be reimbursed. Trial sites will be reimbursed for staff time and consumable costs associated with the conduct of the trial. An agreement with each site will be in place prior to the start of the trial.

#### Audits and inspections

The trial is subject to audit by the LSHTM under their legal obligation as sponsor. Additionally, inspections can be carried out by relevant RECs and other regulatory authorities to ensure adherence to the protocol, GCP and relevant regulations.

### Trial management

#### Trial Management Group

A Trial Management Group (TMG) will be responsible for overseeing the progress of the trial. The day-to-day management of the trial will be coordinated through the LSHTM CTU. The TMG will consist of the Protocol Committee members plus a trial manager, data manager and trial administrator.

The CTU will act on behalf of the sponsor and will be responsible to the TMG to ensure that all of the sponsor’s responsibilities are carried out. The responsibilities include (but are not limited to):

- Reporting to the Trial Steering Committee
- Maintaining the Trial Master File
- Identifying trial sites
- Assessing suitability of trial sites
- Confirming that all approvals are in place before enrolment of participants and release of the trial treatment
- Providing training about the trial, including site initiation
- Providing study materials
- Data management
- 24-h advice and un-blinding service
- Giving collaborators regular information about the progress of the study
- Responding to any questions (e.g. from collaborators) about the trial
- Communicating all important protocol modifications to relevant parties
- Monitoring of the trial
- Ensuring data security and quality and observe data protection laws
- Safety reporting
- Ensuring that the trial is conducted in accordance with the ICH GCP

### Statistical analysis

- publication of trial results.

#### National coordination for each participating country

A national coordinating investigator will be identified for each participating country. They will be responsible for ensuring that all national approvals including those from regulatory agencies, Ethics Committees and relevant import licences are in place before the trial can start in their country. Additionally, they will support the LSHTM CTU with ensuring recruitment is on target, safety reporting to all relevant agencies, and site training and monitoring as required.

#### Protocol development

The Protocol Committee consists of the following investigators who will be responsible for the development of and agreeing the final protocol. Subsequent changes to the final protocol will require the agreement of the TSC:

- Sabaratnam Arulkumaran, Professor Emeritus of Obstetrics and Gynaecology, St. George’s, University of London, UK
- Imelda Bates, Professor, Liverpool School of Tropical Medicine, Liverpool, UK
- Rizwana Chaudhri, Professor, Rawalpindi Medical College, Pakistan (National Coordinating Investigator, Pakistan)
- Bukola Fawole, Professor, University College Hospital, Ibadan, Nigeria (National Coordinating Investigator, Nigeria)
- Katharine Ker, Assistant Professor, Clinical Trials Unit, LSHTM, London, UK
- Ian Roberts, Professor, Clinical Trials Unit, LSHTM, London, UK (co-lead investigator)
- Haleema Shakur-Still, Associate Professor, Clinical Trials Unit, LSHTM, London, UK (co-lead investigator)

#### Independent DMC

The primary responsibility for monitoring the safety of participants in the trial lies with the sponsor. This is overseen by an independent DMC appointed to support the safety monitoring. The composition of the DMC is provided in Additional file 8.

The DMC will review on a regular basis accumulating data from the ongoing trial and advise the TSC regarding the continuing safety of current participants and those yet to be recruited, as well as reviewing the validity and scientific merit of the trial.

The DMC composition, name, title and address of the chairman and of each member, will be given in the DMC Charter which will be in line with that proposed by the DAMOCLES Study Group [38]. Membership includes expertise in the relevant field of study, statistics and research study design.

The DMC Charter includes, but is not limited to, defining:

- The schedule and format of the DMC meetings
- The format for presentation of data
- The method and timing of providing interim reports
- Stopping rules

The DMC is independent from the sponsor, Ethics Committees, regulatory agencies, investigators, Steering Committee membership, clinical care of the trial participants, and any other capacity related to trial operations. The DMC has the responsibility for deciding whether, whilst randomisation is in progress, the un-blinded results (or the un-blinded results for a particular subgroup) should be revealed to the TSC. The DMC Charter states that they will do this if, and only if, two conditions are satisfied: (1) the results provide proof beyond reasonable doubt that treatment is on balance either definitely harmful or definitely favourable for all, or for a particular category of, participants in terms of the major outcome and (2) the results, if revealed, would be expected to substantially change the prescribing patterns of clinicians who are already familiar with any other trial results that exist. Exact criteria for ‘proof beyond reasonable doubt’ are not, and cannot be, specified by a purely mathematical stopping rule, but they are strongly influenced by such rules. The DMC Charter is in agreement with the Peto-Haybittle [39, 40] stopping rule whereby an interim analysis of major endpoint would generally need to involve a difference between treatment and control of at least three standard errors to justify premature disclosure. An interim subgroup analysis would, of course, have to be even more extreme to justify disclosure. This rule has the advantage that the exact number and timing of interim analyses need not be pre-specified. In summary, the stopping rules require extreme differences to justify premature disclosure and involve an appropriate combination of mathematical stopping rules and scientific judgment.

#### Trial Steering Committee (TSC)

The TSC will include independent individuals and members from the TMG. The composition of the TSC is provided in Additional file 9. The role of the TSC is to provide supervision of the trial and to advise the sponsor. In particular, the TSC will concentrate on the progress of the trial, adherence to the protocol, participant safety, and consideration of new information. The TSC must be in agreement with the final protocol and, throughout the trial, will take responsibility for:

- Major decisions such as a need to change the protocol for any reason
- Monitoring and supervising the progress of the trial
- Reviewing relevant information from other sources
- Considering recommendations from the DMC
- Informing and advising the TMG on all aspects of the trial

The TSC includes an experienced obstetrician, clinical trialists, lead investigators, clinical representative from a LMIC, and a lay representative. Face-to-face meetings or teleconferences will be held at regular intervals determined by need, but no less than once a year. A TSC Charter, which will detail how it will conduct its business, will be agreed at the first meeting.

#### Site PI’s responsibilities

Coordination within each participating hospital will be through a site PI whose responsibility will be detailed in an agreement in advance of starting the trial and will include:

- Personally supervising the study at site
- Before and if needed during the trial, obtain all appropriate approval/favourable opinions
- Delegating trial-related responsibilities only to suitably trained and qualified personnel
- Documenting delegation of duties to appropriately qualified persons
- Training relevant medical, midwifery and nursing staff to ensure that they remain aware of the state of the current knowledge, the trial and its procedures (there are wall charts, pocket summaries, training films and PowerPoint presentations to assist with this)
- Agreeing to comply with the final trial protocol and any relevant amendments
- Ensuring that all potentially eligible women are considered promptly for the trial
- Ensuring that consent is obtained in line with local approved procedures
- Ensuring that the data are collected and completed and transmitted to the CTU in a timely manner
- Ensuring that all adverse events are reported promptly to the CTU
- Ensuring that the Investigator’s Study File is up-to-date and complete
- Accounting for trial treatments at their site
- Ensuring that the trial is conducted in accordance with ICH GCP and fulfils all national and local regulatory requirements
- Allowing access to source data, including participants’ medical records for monitoring, audit and inspection
- Being responsible for archiving all original trial documents including medical records, Investigator’s Study File, consent forms and data forms for at least 5 years after the end of the trial

### Publication policy

All publications and presentations relating to the study will be authorised by the lead investigators. Publications will only contained anonymised data. We aim to publish the main results of the WOMAN-2 trial in a peer-reviewed journal under a CC-BY licence. This licence will ensure that the publication is freely available and can be distributed by others as long as they give credit to the original creation. All publications will follow the Consolidated Standards of Reporting Trials

(CONSORT) Statement. Links to publications will be made in all applicable trial registers. The results will be disseminated via the media, trial website and relevant maternal health organisations. The main publication of the trial results will be in the name of the Trial Collaborative Group (WOMAN-2 trial collaborators).

## Discussion

The WOMAN-2 trial will provide reliable evidence for the effects of TXA for preventing postpartum bleeding in women with anaemia. If the WOMAN-2 trial shows that TXA reduces PPH in anaemic women, we would have identified a way of improving the wellbeing of thousands of women world-wide.

## Trial status

The current protocol is version 1.2, dated 10 July 2018. The protocol was approved by the London School of Hygiene and Tropical Medicine’s Ethics Committee (ref: 15194). National ethics and regulatory approvals are in progress in three countries. Patient recruitment is planned to start by January 2019. End of recruitment is planned for January 2021 with end of follow-up in March 2021. Further information is available at http://woman2.lshtm.ac.uk/.

## Additional files

Additional file 1: Standard Protocol Items: Recommendation for Interventional Trials (SPIRIT) Checklist. (DOC 123 kb)

Additional file 2: World Health Organisation (WHO) trial registration dataset. (DOCX 17 kb)

Additional file 3: Brief information sheet. (DOCX 26 kb)

Additional file 4: Overview of consent process. (DOCX 52 kb)

Additional file 5: Patient information. (DOCX 68 kb)

Additional file 6: Overview of safety reporting. (DOCX 97 kb)

Additional file 7: Contact details. (DOCX 23 kb)

Additional file 8: Data Management Committee (DMC) membership. (DOCX 23 kb)

Additional file 9: Trial Steering Committee (TSC) membership. (DOCX 23 kb)

## Abbreviations

AE: Adverse event
APPT: Activated partial thromboplastin time
AR: Adverse reaction
CONSORT: Consolidated Standards of Reporting Trials
CRF: Case Report Form
CTU: Clinical Trials Unit
DAL: Drug Accountability Log
DMC: Data Monitoring Committee
DVT: Deep vein thrombosis
GCP: Good Clinical Practice
GMP: Good Manufacturing Practice
Hb: Haemoglobin
HRQoL: Health-related quality of life
IB: Investigator’s Brochure
ICH GCP: International Conference on Harmonisation of Good Clinical Practice
ICMJE: International Committee for Medical Journal Editors
IEC: Independent Ethics Committee
IMP: Investigational Medicinal Product
IRB: Institutional Review Board
LMICs: Low- and middle-income countries
LSHTM: London School of Hygiene and Tropical Medicine
PCV: Packed cell volume
PI: Principal investigator
PPH: Postpartum haemorrhage
QP: Qualified person
REC: Research Ethics Committee
RR: Relative Risk
SAE: Serious adverse event
SAP: Statistical Analysis Plan
SAR: Serious adverse reaction
SUSAR: Suspected unexpected serious adverse reaction
TMG: Trial Management Group
TPA: Tissue plasminogen activator
TSC: Trial Steering Committee
TXA: Tranexamic acid
UK: United Kingdom
WHO: World Health Organisation

## References

[1] Calvert C, Thomas SL, Ronsmans C, Wagner KS, Adler AJ, Filippi V (2012). Identifying regional variation in the prevalence of postpartum haemorrhage: a systematic review and meta-analysis. PLoS One.

[2] Carroli G, Cuesta C, Abalos E, Gulmezoglu A. Epidemiology of postpartum haemorrhage: a systematic review. Best Pract Res Clin Obstet Gynaecol. 2008;22(6):999–1012. 10.1016/j.bpobgyn.2008.08.004 18819848

[3] WHO UaTWB (2012). Trends in maternal mortality: 1990 to 2010 – WHO, UNICEF, UNFPA and The World Bank estimates.

[4] Say L, Pattinson R, Gulmezoglu A. WHO systematic review of maternal morbidity and mortality: the prevalence of severe acute maternal morbidity (near miss). BioMed Central Reproductive Health. 2004;1(1):3. 10.1186/1742-4755-1-3 PMC516581 15357863

[5] Thompson JF, Heal LJ, Roberts CL, Ellwood DA (2010). Women’s breastfeeding experiences following a significant primary postpartum haemorrhage: a multicentre cohort study. Int Breastfeed J.

[6] Ricbourg A, Gosme C, Gayat E, Ventre C, Barranger E, Mebazaa A (2015). Emotional impact of severe post-partum haemorrhage on women and their partners: an observational, case-matched, prospective, single-centre pilot study. Eur J Obstet Gynecol Reprod Biol.

[7] Dhingra N. Making Safe Blood Available in Africa. 2006. http://www.who.int/bloodsafety/makingsafebloodavailableinafricastatement.pdf. Accessed 14 Dec 2018.

[8] Nair M, Choudhry MK, Choudhry SS, Kakoty SD, Sarma UC, Webster P, Knight M, on behalf of the IndOSS-Assam Steering Committee. Association between maternal anaemia and pregnancy outcomes: a cohort study in Assam, India. BMJ Global Health. 2016;1:e000026. 10.1136/bmjgh-2015-000026. 10.1136/bmjgh-2015-000026 PMC5321311 28588921

[9] Sheldon WR, Blum J, Vogel JP, Souza JP, Gulmezoglu AM, Winikoff B, Maternal WHOMSo, Newborn Health Research Network (2014). Postpartum haemorrhage management, risks, and maternal outcomes: findings from the World Health Organization Multicountry Survey on Maternal and Newborn Health. BJOG.

[10] Geller S, Adams M, Kelly P, Kodkany B, Derman R (2006). Postpartum hemorrhage in resource poor-settings. Int J Gynecol Obstet.

[11] WHO. Global prevalence of anaemia in 2011. 2015. http://apps.who.int/iris/bitstream/10665/177094/1/9789241564960_eng.pdf. Accessed 14 Dec 2018.

[12] Stevens GA, Finucane MM, De-Regil LM, Paciorek CJ, Flaxman SR, Branca F, Pena-Rosas JP, Bhutta ZA, Ezzati M, Nutrition Impact Model Study Group (2013). Global, regional, and national trends in haemoglobin concentration and prevalence of total and severe anaemia in children and pregnant and non-pregnant women for 1995-2011: a systematic analysis of population-representative data. Lancet Glob Health.

[13] Woman Trial Collaborators (2017). Effect of early tranexamic acid administration on mortality, hysterectomy, and other morbidities in women with post-partum haemorrhage (WOMAN): an international, randomised, double-blind, placebo-controlled trial. Lancet.

[14] WHO. Haemoglobin concentrations for the diagnosis of anaemia and assessment of severity. Vitamin and Mineral Nutrition Information System. Geneva, World Health Organization, 2011 (WHO/NMH/NHD/MNM/11.1) http://www.who.int/vmnis/indicators/haemoglobin.pdf. Accessed 23 Feb 2018.

[15] Ker K, Prieto-Merino D, Roberts I (2013). Systematic review, meta-analysis and meta-regression of the effect of tranexamic acid on surgical blood loss. Br J Surg.

[16] Novikova N, Hofmeyr GJ (2010). Tranexamic acid for preventing postpartum haemorrhage. Cochrane Database Syst Rev.

[17] Ker K, Shakur H, Roberts I (2016). Does tranexamic acid prevent postpartum haemorrhage? A systematic review of randomised controlled trials. BJOG.

[18] Sentilhes L, Winer N, Azria E, Sénat M, Le Ray C, Vardon D, Perrotin F, Desbrière R, Fuchs F, Kayem G (2018). Tranexamic acid for the prevention of postpartum hemorrhage after vaginal delivery: the TRAAP trial. Am J Obstet Gynecol.

[19] Galambosi PJ, Gissler M, Kaaja RJ, Ulander VM (2017). Incidence and risk factors of venous thromboembolism during postpartum period: a population-based cohort-study. Acta Obstet Gynecol Scand.

[20] James AH, Jamison MG, Brancazio LR, Myers ER (2006). Venous thromboembolism during pregnancy and the postpartum period: incidence, risk factors, and mortality. Am J Obstet Gynecol.

[21] Eriksson K, Nilsson (1971). Tranexamic acid in human milk after oral administration of cyklokapron to lactating women.

[22] Pharmacia. Cyklokapron. ABPI Compendium of data sheets and summaries of product characteristics. London: Datapharm Publications Ltd. 1998–99.

[23] Chan AW, Tetzlaff JM, Gotzsche PC, Altman DG, Mann H, Berlin JA, Dickersin K, Hrobjartsson A, Schulz KF, Parulekar WR (2013). SPIRIT 2013 explanation and elaboration: guidance for protocols of clinical trials. BMJ.

[24] Maanongun MT, Daru PH, Pam VC, Swende TZ, Ojabo AO, Eka PO (2016). Labour outcome in patients admitted in the second stage of labour at Jos University Teaching Hospital, Jos, Nigeria. Trop J Obstet Gynaecol.

[25] Concordia International. Summary of Product Characteristics – Tranexamic acid 500 mg/5 ml solution for injection [updated 21 Feb 2017]. https://www.medicines.org.uk/emc/product/3374. Accessed 14 Dec 2018.

[26] WOMAN Trial Collaborators. Effect of early administration of tranexamic acid on mortality, hysterectomy, other morbidities in women with postpartum haemorrhage (The WOMAN trial): a randomised, placebo-controlled trial. Lancet, 2017. in press 10.1016/S0140-6736(17)30638-4 PMC5446563 28456509

[27] The CRASH-2 Collaborators (2010). Effects of tranexamic acid on death, vascular occlusive events, and blood transfusion in trauma patients with significant haemorrhage (CRASH-2): a randomised, placebo-controlled trial. Lancet.

[28] The CRASH-2 Collaborators (2011). The importance of early treatment with tranexamic acid in bleeding trauma patients: an exploratory analysis of the CRASH-2 randomised controlled trial. Lancet.

[29] Ker K, Edwards P, Perel P, Shakur H, Roberts I (2012). Effect of tranexamic acid on surgical bleeding: systematic review and cumulative meta-analysis. BMJ.

[30] Summary of Product Characteristics for Cyklokapron: http://www.medicines.org.uk/emc/medicine/16512/SPC/Cyklokapron+Tablets/. Accessed 31 Mar 2017.

[31] Keyl C, Uhl R, Beyersdorf F, Stampf S, Lehane C, Wiesenack C, Trenk D (2011). High-dose tranexamic acid is related to increased risk of generalized seizures after aortic valve replacement. Eur J Cardiothorac Surg.

[32] Manji RA, Grocott HP, Leake J, Ariano RE, Manji JS, Menkis AH, Jacobsohn E (2012). Seizures following cardiac surgery: the impact of tranexamic acid and other risk factors. Can J Anesth.

[33] Murkin JM, Falter F, Granton J, Young B, Burt C, Chu M (2010). High-dose tranexamic acid is associated with nonischemic clinical seizures in cardiac surgical patients. Anesth Analg.

[34] Heit JA, Kobbervig CE, James AH, Petterson TM, Bailey KR, Melton LJ (2005). Trends in the incidence of venous thromboembolism during pregnancy or postpartum: a 30-year population-based study. Ann Intern Med.

[35] Pealing L, Perel P, Prieto-Merino D, Roberts I, Collaborators C-T (2012). Risk factors for vascular occlusive events and death due to bleeding in trauma patients; an analysis of the CRASH-2 cohort. PLoS One.

[36] Gilad O, Merlob P, Stahl B, Klinger G (2014). Outcome following tranexamic acid exposure during breastfeeding. Breastfeed Med.

[37] Holland AE, Spruit MA, Troosters T, Puhan MA, Pepin V, Saey D, McCormack MC, Carlin BW, Sciurba FC, Pitta F (2014). An official European Respiratory Society/American Thoracic Society technical standard: field walking tests in chronic respiratory disease. Eur Respir J.

[38] DAMOCLES study group (2005). A proposed charter for clinical trial data monitoring committees: helping them to do their job well. Lancet.

[39] Peto R, Pike MC, Armitage P, Breslow NE, Cox DR, Howard SV, Mantel N, McPherson K, Peto J, Smith PG (1977). Design and analysis of randomized clinical trials requiring prolonged observation of each patient. II analysis and examples. Br J Cancer.

[40] Haybittle JL (1971). Repeated assessment of results in clinical trials of cancer treatment. Br J Radiol.

