# Supplementary material for: Tranexamic acid for the prevention of postpartum bleeding in women with anaemia: study protocol for an international, randomised, double-blind, placebo-controlled trial
Source: Trials. 2018 Dec 29;19:712. doi: 10.1186/s13063-018-3081-x (PMC6311062; doi:10.1186/s13063-018-3081-x)
Supplement: Supplementary file 2 — World Health Organisation (WHO) trial registration dataset. (DOCX 17 kb) [file 13063_2018_3081_MOESM2_ESM.docx]

**WHO Trial Registration Data Set**

| 1 | Primary Registry and Trial Identifying Number | ISRCTN62396133 |
| --- | --- | --- |
| 2 | Date of Registration in Primary Registry | 7 December 2017 |
| 3 | Secondary Identifying Numbers | Sponsor ref: LSHTM 2018-KEP-007  LSHTM Ethics ref: 15194  ClinicalTrials.gov Identifier: NCT03475342 |
| 4 | Source(s) of Monetary or Material Support | Wellcome  Bill & Melinda Gates Foundation |
| 5 | Primary Sponsor | London School of Hygiene & Tropical Medicine |
| 6 | Secondary Sponsor(s) | n/a |
| 7 | Contact for Public Queries | Clinical Trials Unit, Keppel Street, London, WC1E 7HT, UK +44(0)20 7299 4684  [woman2@lshtm.ac.uk](mailto:woman2@lshtm.ac.uk) |
| 8 | Contact for Scientific Queries | Haleema Shakur-Still, Clinical Trials Unit, Keppel Street, London, WC1E 7HT, UK +44(0)20 7299 4684  [woman2@lshtm.ac.uk](mailto:woman2@lshtm.ac.uk) |
| 9 | Public Title | WOrld Maternal ANtifibrinolytic trial (WOMAN-2) |
| 10 | Scientific Title | Tranexamic acid for the prevention of postpartum bleeding in women with anaemia: an international, randomised, double-blind, placebo controlled trial |
| 11 | Countries of Recruitment | Nigeria, Pakistan, Uganda |
| 12 | Health Condition(s) or Problem(s) Studied | Prevention of postpartum haemorrhage in women with moderate or severe anaemia having given birth vaginally |
| 13 | Intervention(s) | Active Comparator: Tranexamic acid, one intravenous injection of tranexamic acid. Total dose 1 gram (10mL). Placebo Comparator: One Injection of 10 mL Sodium Chloride (0.9%). |
| 14 | Key Inclusion and Exclusion Criteria | Inclusion criteria: Women with moderate or severe anaemia (haemoglobin level <100 g/L or packed cell volume <30%), who have given birth vaginally and for who the responsible clinician is substantially uncertain whether to use TXA.  Exclusion criteria: Women who are not legally adult (<18 years) and permission not provided by a guardian; women with a known allergy to TXA or its excipients; women who develop PPH before umbilical cord is clamped/cut. |
| 15 | Study Type | Type of study: interventional  Method of allocation: randomised  Masking: double-blind  Assignment: parallel  Phase: 3  Sequence generation and allocation concealment: Randomisation is balanced by centre with a 1:1 allocation sequence in blocks of size 20 (corresponding to a box of trial treatment packs) so that each box contains 10 packs of TXA and 10 of placebo in random order. Each woman will be randomised by selecting the lowest numbered, intact pack from a box of 20 sealed treatment packs. Allocation concealment is ensured by the use of numbered but otherwise identical treatment packs. The randomisation list will be generated by an independent randomisation system service provider and sent to the clinical trial supplies company responsible for preparing the treatment packs. |
| 16 | Date of First Enrolment | Anticipated date of enrolment of the first participant: January 2019 |
| 17 | Sample Size | Planned sample size: 10,000 |
| 18 | Recruitment Status | Recruitment status of this trial: Pending |
| 19 | Primary Outcome(s) | Outcome name: clinical diagnosis of primary postpartum haemorrhage  Method of measurement: This may be an estimated blood loss of more than 500 mL or any blood loss sufficient to compromise haemodynamic stability within 24 hours of administration of trial medication. Haemodynamic instability is based on clinical judgement and assessed using clinical signs (low systolic blood pressure, tachycardia, reduced urine output). The cause of PPH will be described.  Timepoint: Within 24 hours of trial treatment. |
| 20 | Key Secondary Outcomes | Outcome name: Postpartum blood loss  Method of measurement: clinical assessment  Timepoint: 24 hours after trial treatment or at discharge from hospital whichever is earlier  Outcome name: Haemaglobin  Method of measurement: Haemacue (Point of care test)  Timepoint: 24 hours after trial treatment or at discharge from hospital whichever is earlier  Outcome name: Haemodynamic instability  Method of measurement: based on clinical signs e.g. low blood pressure, tachycardia, reduced urine output requiring intervention e.g. intravenous fluid  Timepoint: 24 hours after trial treatment or at discharge from hospital whichever is earlier  Outcome name: Shock index  Method of measurement: using lowest recorded heart rate/systolic blood pressure  Timepoint: 24 hours after trial treatment or at discharge from hospital whichever is earlier  Outcome name: Symptoms of anaemia  Method of measurement: questionnaire  Timepoint: at 42 days or discharge from hospital whichever is earlier  Outcome name: Quality of life, including overall wellbeing, ability to care for herself and her baby, breastfeeding (time to first feed, ability to sustain breastfeeding)  Method of measurement: questionnaire  Timepoint: at 42 days or discharge from hospital whichever is earlier  Outcome name: Expected side effects of trial medication  Method of measurement: patient self reports and medical records  Timepoint: at 42 days or discharge from hospital whichever is earlier  Outcome name: Exercise tolerance  Method of measurement: 6 minute walk test  Timepoint: at 42 days or discharge from hospital whichever is earlier  Outcome name: Interventions to control primary postpartum haemorrhage  Method of measurement: medical records  Timepoint: at 42 days or discharge from hospital whichever is earlier  Outcome name: Receipt of blood transfusion  Method of measurement: medical records  Timepoint: at 42 days or discharge from hospital whichever is earlier  Outcome name: Vascular occlusive events  Method of measurement: medical records  Timepoint: at 42 days or discharge from hospital whichever is earlier  Outcome name: Organ dysfunction  Method of measurement: medical records  Timepoint: at 42 days or discharge from hospital whichever is earlier  Outcome name: Sepsis  Method of measurement: medical records  Timepoint: at 42 days or discharge from hospital whichever is earlier  Outcome name: Hospital death  Method of measurement: medical records  Timepoint: at 42 days or discharge from hospital whichever is earlier  Outcome name: Length of hospital stay  Method of measurement: medical records  Timepoint: at 42 days or discharge from hospital whichever is earlier  Outcome name: Admission to and time spent in higher level facility  Method of measurement: medical records  Timepoint: at 42 days or discharge from hospital whichever is earlier  Outcome name: Status of baby/babies  Method of measurement: medical records  Timepoint: at 42 days or discharge from hospital whichever is earlier  Outcome name: Thromboembolic events in breastfed babies  Method of measurement: medical records  Timepoint: at 42 days or discharge from hospital whichever is earlier  Outcome name: Adverse events  Method of measurement: medical records  Timepoint: at 42 days or discharge from hospital whichever is earlier |
| 21 | Ethics Review | Status: Approved  Date of approval: 10 May 2018  Name and contact details of Ethics committee(s): Professor John DH Porter (Chair), Observational / Interventions Research Ethics Committee, London School of Hygiene & Tropical Medicine, Keppel St, London EC1E 7HT. [ethics@lshtm.ac.uk](mailto:ethics@lshtm.ac.uk) |
| 22 | Completion date | Date of study completion: 31/01/2022 |
| 23 | Summary Results | n/a – trial in progress |
| 24 | IPD sharing statement | Plan to share IPD: Yes  Plan description: The LSHTM CTU is committed to sharing its clinical study data for additional, ethical research with justified scientific objectives. Until all planned analyses are completed by the LSHTM CTU, data will be shared through a controlled access approach whereby researchers can make formal applications for data sharing. Afterwards, totally anonymised data will be shared via the LSHTM CTU data sharing platform at freebird.lshtm.ac.uk. |
