# Supplementary material for: Tranexamic acid for the prevention of postpartum bleeding in women with anaemia: study protocol for an international, randomised, double-blind, placebo-controlled trial
Source: Trials. 2018 Dec 29;19:712. doi: 10.1186/s13063-018-3081-x (PMC6311062; doi:10.1186/s13063-018-3081-x)
Supplement: Supplementary file 3 — Brief information sheet. (DOCX 26 kb) [file 13063_2018_3081_MOESM3_ESM.docx]

**BRIEF STUDY INFORMATION SHEET**

**THE WOMAN-2 TRIAL**

This hospital is involved in a research study to try and find ways of preventing severe bleeding in anaemic women after they have given birth.

Thank you for taking the time to read this leaflet.

**What is the WOMAN-2 study?**

The WOMAN-2 trial is a research study to see whether using a drug called tranexamic acid will stop women with anaemia from developing severe bleeding after having a baby (postpartum haemorrhage). This study will involve about 10,000 women giving birth in hospitals in Africa and Asia.

**What is postpartum haemorrhage?**

Most women who give birth have no problems during or after the delivery of their baby. Following every birth there will be a small amount of bleeding from the mother – this is normal and usually nothing to worry about. However, occasionally after the baby is born there is much more bleeding. This extra bleeding is called postpartum haemorrhage (PPH). When this happens the doctors, nurses and midwives will do everything they can to stop the bleeding, because if too much blood is lost the mother may become very unwell.

Low levels of iron in the blood (a condition known as anaemia) is common among pregnant women. Having anaemia increases the chances of having a PPH and women with anaemia who have a PPH tend to suffer more afterwards than women without anaemia. For this reason, we want to find a way to reduce the chances of women with anaemia from having severe bleeding after childbirth.

There are treatments that can be given to women to prevent PPH but some women will still have severe bleeding despite this. Also, there are treatments to help control PPH when it starts. But it is not always possible to give these treatments quickly enough to stop some women from becoming very unwell. It would be better if we could find a way of preventing PPH from happening in the first place.

**What is tranexamic acid?**

Tranexamic acid (TXA) is a drug that reduces bleeding. It is not a new drug and it is widely used to reduce bleeding after operations and to treat bleeding after injury. It is also one of the treatments that can be given to women with PPH to reduce bleeding.

**What does the study involve?**

When you are about to have your baby, you may be asked to take part in the study. You will only be asked if you plan to have a vaginal birth and if your blood test shows that you have anaemia. The study involves you agreeing to take part and if you agree, you will receive an injection of either the TXA or a placebo (a dummy drug) directly into a vein, immediately after your baby is born and the umbilical cord is cut or clamped. We will collect information on whether or not you develop a PPH and your progress while in hospital. Before you leave hospital, we will ask you some questions about how you and your baby are. We will also ask you to do a walking test, to see how far you can walk in six minutes.

Being part of this study will not interfere with any other treatment or with how you plan to have your baby.

If there is time before you give birth and you are able to absorb the full information about the trial, we will provide this to you and ask you to sign a consent form.

Otherwise, we will tell you that this is a research study, that you do not have to take part if you do not wish to and that if you say no it will not interfere with any care you receive from this hospital. We will explain the information contained above and you can ask any questions and if you say yes, we will start the study by collecting some information from your medical records to make sure you are suitable. If you are suitable, the study drug will be given to you. We will collect information from your medical records afterwards on how you are doing. Immediately you are well enough, we will give you the full study information and get your full consent to continue the study.

If you want more information about the study, the study coordinators at this hospital can be contacted on:

Name

Address

Phone

Email

The study is organised by the London School of Hygiene & Tropical Medicine (University of London) and is supervised in [Country] by xxxx. You can also contact them directly for information about the trial.

Name of NCC

Address

Phone

Email
