# Supplementary material for: Tranexamic acid for the prevention of postpartum bleeding in women with anaemia: study protocol for an international, randomised, double-blind, placebo-controlled trial
Source: Trials. 2018 Dec 29;19:712. doi: 10.1186/s13063-018-3081-x (PMC6311062; doi:10.1186/s13063-018-3081-x)
Supplement: Supplementary file 4 — Overview of consent process. (DOCX 52 kb) [file 13063_2018_3081_MOESM4_ESM.docx]

## Consent procedure overview

Woman in active labour, plans to give birth vaginally with Hb <100 g/L or PCV <30%. No indication or contraindication to TXA.

- Researcher obtaining consent must sign the consent form
- Consent process used should be documented in woman’s medical notes
- File original consent form in Investigator’s Study File
- Give copy of signed form to woman
- File one signed copy in the woman’s medical notes

YES

YES

Willing to be considered for inclusion in the trial and

fully competent to give valid informed consent?

Willing to be considered for inclusion in the trial but unable to give fully informed consent?

YES

YES

- Full information given to woman and written informed consent obtained by researcher
- Baseline data collected
- Eligibility confirmed
- Woman randomised and treatment administered immediately after eligibility confirmed
- Brief verbal information given to woman
- Verbal agreement obtained by researcher in the presence of an independent witness
- Baseline data collected
- Eligibility confirmed
- Woman randomised and treatment administered immediately after eligibility confirmed
- When women regains capacity, obtain written consent for continuing in the study

**If woman is <18 years old:**

- Consent must be witnessed by an appropriate responsible person who must also sign the consent form

**If woman is unable to read or write:**

- Explain the trial in the presence of an independent witness
- Obtain mark (e.g. thumbprint) from woman
- Independent witness must sign the form

**In all cases:**

- Researcher obtaining consent must sign the consent form
- File original consent form in Investigator’s Site File
- Give copy of signed form to woman
- File one signed copy in the woman’s medical notes
- Consent process used should be documented in woman’s medical notes
