# Supplementary material for: Tranexamic acid for the prevention of postpartum bleeding in women with anaemia: study protocol for an international, randomised, double-blind, placebo-controlled trial
Source: Trials. 2018 Dec 29;19:712. doi: 10.1186/s13063-018-3081-x (PMC6311062; doi:10.1186/s13063-018-3081-x)
Supplement: Supplementary file 5 — Patient information. (DOCX 68 kb) [file 13063_2018_3081_MOESM5_ESM.docx]

[HOSPITAL CONTACT DETAILS]


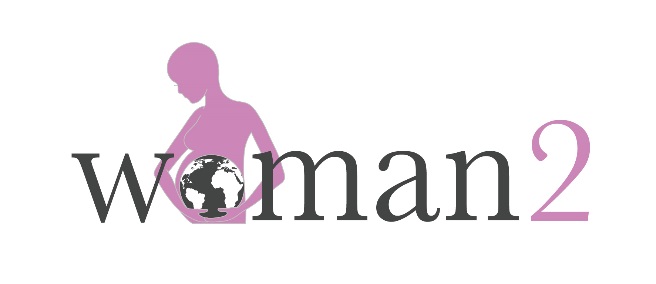


Tranexamic acid for the prevention of postpartum bleeding in women with anaemia:

an international, randomised, double-blind, placebo controlled trial.

**STUDY INFORMATION FOR PARTICIPANTS**

(GENERIC VERSION)

**Photo by Paolo Patruno Photography/IDEAS 2015**.

| **We invite you to take part in a research study called WOMAN-2**   - Before you decide to take part or not, we would like you to know why the study is being done and what it will involve. - Please read this information. You can talk to others about the study if you wish. - You can ask the doctor or midwife looking after you as many questions about the study as you like before deciding to take part or not. - It is up to you to decide to take part in this study or not. If you choose not to take part, your doctors and midwives will give you the usual care given at this hospital. |  | **Contents**   1. What is the study for? 2. Why are you asking me to take part? 3. What will happen if I take part? 4. How long will I be in the study? 5. Will I benefit from taking part? 6. Could I be harmed by taking part? 7. Can I change my mind about taking part? 8. What happens afterwards? 9. What information do we keep private? 10. Who is doing this study? 11. Who has reviewed the study? 12. What if there is a problem? 13. What else do I need to know? |
| --- | --- | --- |

1. **What is the study for?**

This study will find out if giving women a drug called tranexamic acid, can reduce how much they bleed after giving birth. It is very common to bleed after giving birth and for most women it does not cause any problems. But some women lose a lot of blood which can make them very unwell. Some women also have a condition called anaemia, which means that their blood does not work as well as it should. It is very important that women who have anaemia do not bleed too much after giving birth, as even small amounts of bleeding can make them very unwell.

There is a drug called tranexamic acid that can help bleeding to stop. Giving tranexamic acid to women who already have a large bleed after giving birth, helps them to recover. But it would be better if we could stop women from having a large bleed in the first place, especially if they have anaemia. So we want to find out if giving tranexamic acid to women who have anaemia, straight after they have given birth, stops them from bleeding too much.

We hope that tranexamic acid will reduce the amount of blood women lose and so they will feel better than women who receive the dummy drug, but we don’t yet know if it will. This is why we are doing the study.

1. **Why are you asking me to take part?**

We are asking you to take part because you are expected to have a vaginal birth and you have anaemia.

We are giving this information to you and asking you to take part now, so that if you agree the study team at this hospital can include you in the study. If after you have agreed to take part, your doctor thinks that it would be better for you to have an operation to have your baby (called a caesarean section) you will not be included in the study.

**You will not be able to take part if:**

- You do not deliver your baby vaginally
- You are less than 18 years old and your guardian has said you cannot be in the study or you do not have a guardian available
- You are allergic to the study drug or what it is mixed with
- You develop severe bleeding before the umbilical cord is clamped or cut
- Your doctor thinks tranexamic acid would not be good for your health
- Your doctor thinks you should receive tranexamic acid as it would be good for your health

About 10,000 women across Africa and Asia will be taking part in this study. It is up to you to decide if you wish to take part or not.

1. **What will happen if I take part?**

Taking part in the study will not change how you give birth and you will get all the usual care given to women giving birth in your hospital.

We will ask you to fill in a form to say that you are willing to take part. A person from the study team at your hospital will then write down some information about you and your labour. As soon as your baby is born and the umbilical cord is cut, you will be given an injection of either tranexamic acid or placebo (a dummy drug). Which of the two injections each women is given is decided randomly and each has an equal chance receiving either injection. The study drug and the placebo look the same, so the women and their doctors will not know which drug they were given.

The day after you give birth, we will collect some information from your medical notes. We will also take a very small sample of blood from your finger to check your anaemia. Before you leave hospital, we will ask you some questions about how you and your baby are. We will also ask you to do a walking test, to see how far you can walk in six minutes. You can decide at the time if you feel able to do this test or not. You can stop or rest at any time during the test. It will take about 30 minutes to answer these questions and do the walking test.

1. **How long will I be in this study?**

You will be in the study until you leave hospital, or for six weeks after you had your baby, whichever is sooner. If after leaving hospital and within six weeks of giving birth, you become ill, please let the doctor named on this form know.

1. **What are the benefits of taking part in this study?**

We do not know if taking part in this study will help you. What we learn from this study will help doctors care for woman at risk of having a large bleed after giving birth in the future.

1. **Could I be harmed by taking part?**

Tranexamic acid is not a new drug and it is often used to treat people with other types of bleeding, such as when having an operation or after being injured in an accident. Other studies suggest that it doesn’t have any serious side effects. Sometimes it can cause nausea, vomiting, and diarrhoea. A very small amount of tranexamic acid can pass into breast milk. Other studies have not found any harmful effects in babies who were breastfed by mothers who were given tranexamic acid. Your doctor will watch you and your baby, and give you the best available care if there are any problems. They will also tell the people running the study if there are any problems.

To check you anaemia we will need to take a small sample of blood from your finger. You may feel discomfort or pain when your finger is pricked.

Before you leave the hospital, we will ask you questions about how you are feeling. These questions may bring up some upsetting feelings for you. If you don’t want to answer any of the questions, you do not have to. If you notice that you feel sad or worried, we will ask your doctor to see you to help decide the best way to help you.

1. **Can I change my mind about taking part?**

Yes. You can stop taking part in the study, at any time. You just need to say something like, *“I’ve decided I don’t want to be in this study now”*. Your doctor and the hospital staff will still care for you in the usual way. If you have any medical problems after you stop taking part, we ask that you still tell us about them.

1. **What happens afterwards?**

We will give you a card with the contact details of the study doctor at this hospital. Please keep this card safe. If after you leave hospital you become ill within six weeks of having your baby, please contact the study doctor listed on the card. Also, please show this card to anyone who treats you for any illness.

If you would like to have a copy of the final results of this study, please let the study doctor know and s/he will make sure you receive a copy when the results are published.

You can also visit the study website to keep up to date with the progress of the study: [insert website]

1. **What information do we keep private?**

We will keep all information collected about you and your baby private and stored securely. The only people allowed to look at the information will be the staff who are running the trial at the London Coordinating Centre and the Coordinating Centre in your country [Name], as well as the regulatory authorities who check that the study is being carried out correctly. The London Coordinating Centre may want to collect or copy some study information which will have your name on it such as the signed Consent Form. These will be destroyed or your personal details removed immediately after use.

We will publish the study results in medical journals so that other doctors and midwives can learn from them. We will not include your personal information in any study reports, so you will not be able to be identified. The study team may share data from the study with other researchers and the public, but your personal information will not be included.

1. **Who is doing this study? Who can I contact about any questions, or if I have a problem?**

The study is run by a team of researchers at the London School of Hygiene & Tropical Medicine (University of London) in the United Kingdom.

If you have any questions or concerns about the study, you should ask to speak with the study team who will do their best to answer your questions. You can contact the doctor in charge of the trial at this hospital at:

| Name |  |
| --- | --- |
| Address: |  |
| Telephone: |  |
| Email: |  |

If you wish to complain formally, you can do this through the hospital’s complaints procedure. Please ask the study doctors or midwives for details.

1. **Who has reviewed the study?**

To look after your interests, this study has been carefully checked by an independent group of people called a Research Ethics Committee [Name]. They agreed that it is okay for us to do this study.

1. **What if there is a problem?**

If something goes wrong and you are harmed during the study, the London School of Hygiene & Tropical Medicine would be responsible for claims for any non-negligent harm.

1. **What else do I need to know?**

The study is organised by London School of Hygiene and Tropical Medicine (University of London, UK) and funded by the Wellcome Trust (UK) and the Bill and Melinda Gates Foundation (USA). None of these institutions are the makers of tranexamic acid.

If you agree to take part, you will sign a separate consent form. We will give you a copy of your consent form and this information sheet.

The study treatment is free. It will not cost you any money to take part in this study.

If you return to hospital for any medical problem associated with the study, we will pay your travel costs.

CONSENT FORM

**THE WOMAN-2 TRIAL**

**Title of research**: Tranexamic acid for the prevention of postpartum bleeding in women with anaemia: an international, randomised, double-blind, placebo controlled trial

| Site ID Number |  | Name of Site Principal Investigator |  |
| --- | --- | --- | --- |
| Participant Hospital ID number |  | Screening ID Number |  |
| Name of Participant |  | | |

**Statement of person giving consent:**

1. I confirm that I have read/have had read to me the information sheet for the above study and it was in a language I understand.
2. I have discussed with the doctor to my satisfaction and I have had the opportunity to ask questions.
3. I understand that my participation is voluntary. I have been given enough information about the research study to judge that I want to take part in it.
4. I am free to withdraw at any time, without giving any reason and without my medical care or legal rights being affected.
5. I understand that I will be given a copy of this consent form and the additional information sheet to keep for myself.
6. I understand that sections of my medical notes and those of my baby/ies may be looked at by responsible individuals involved in the study. I give permission for these individuals to have access to these records.
7. I understand that my data (with all personal information removed) will be made freely available for researchers.
8. I give permission for a copy of this consent form, which contains my personal information, to be made available to the Trial Coordinating Centre in London for monitoring purposes only.
9. I agree to take part in the above study, the WOMAN-2 trial.

_______________________________ ________________ ________________________________

Name of woman Date Signature / Thumbprint or other mark (if unable to sign

_____________________________ ________________ ________________________________

Name of witness/guardian Date Signature

*(A witness is needed if a patient cannot read or write and a Guardian signature is needed if a participant is less than 18 years old)*

**Statement of person obtaining informed consent:**

I have fully explained this research to this participant and have given sufficient information, including about risks and benefits, to make an informed decision.

___________________________ ________________ ________________________________

Name Date Signature
