# Supplementary figures and images for: Tranexamic acid for the prevention of postpartum bleeding in women with anaemia: study protocol for an international, randomised, double-blind, placebo-controlled trial
Source: Trials. 2018 Dec 29;19:712. doi: 10.1186/s13063-018-3081-x (PMC6311062; doi:10.1186/s13063-018-3081-x)

**Safety Reporting Overview**


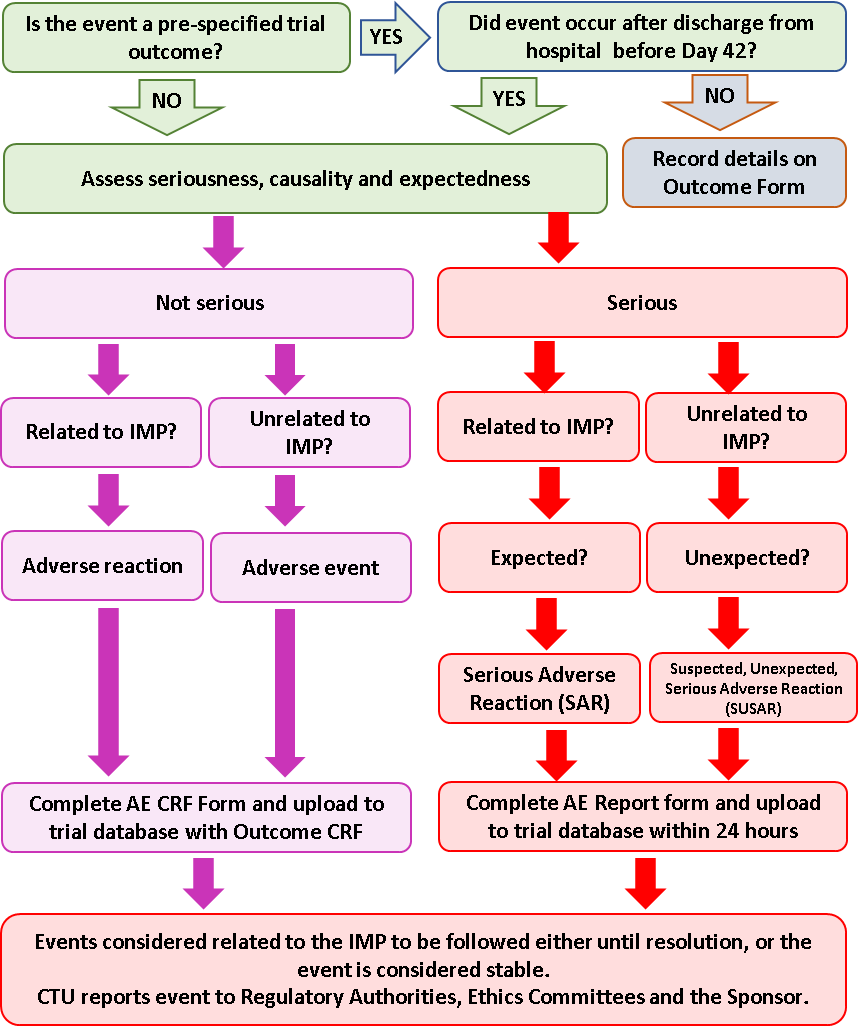

Supplement: Supplementary file 6 — Overview of safety reporting. (DOCX 97 kb) [file 13063_2018_3081_MOESM6_ESM.docx]
