# Supplementary material for: Tranexamic acid for the prevention of postpartum bleeding in women with anaemia: study protocol for an international, randomised, double-blind, placebo-controlled trial
Source: Trials. 2018 Dec 29;19:712. doi: 10.1186/s13063-018-3081-x (PMC6311062; doi:10.1186/s13063-018-3081-x)
Supplement: Supplementary file 7 — Contact details. (DOCX 23 kb) [file 13063_2018_3081_MOESM7_ESM.docx]

## Main Contacts

| CLINICAL TRIALS UNIT - LSHTM WOMAN-2 Trial  Clinical Trials Unit  London School of Hygiene & Tropical Medicine  Keppel Street  London  WC1E 7HT, UK  Tel +44(0)20 7299 4684  Fax +44(0)20 7299 4663  Email: woman2@lshtm.ac.uk  Web: woman2trial.lshtm.ac.uk | SPONSOR The London School of Hygiene & Tropical Medicine is the main research sponsor for this study. For further information regarding the sponsorship conditions, please contact:  Research Governance and Integrity Office  London School of Hygiene & Tropical Medicine  Keppel Street  London  WC1E 7HT, UK  Tel: +44 20 7927 2626  Email: [RGIO@lshtm.ac.uk](mailto:RGIO@lshtm.ac.uk) |
| --- | --- |
| CLINICAL TRIALS UNIT – NIGERIA COMUI-LSHTM Research Collaboration Centre  College of Medicine  University of Ibadan  Queen Elizabeth Road  Ibadan, Nigeria  Tel: +44(0)20 7958 8517  Email: nigeria.woman2@lshtm-ctu.org | EMERGENCY TELEPHONE CONTACT This emergency number is to be used only in the event urgent unblinding of the trial treatment or if advice for reporting an adverse event is needed:  Tel: +44(0)7768 707500 |
| CLINICAL TRIALS UNIT – PAKISTAN RMC-LSHTM Research Collaboration Centre  Room No 199  Department of Obstetrics and Gynaecology  Unit 1, Holy Family Hospital, Said Pur Road  Rawalpindi 46000  Pakistan  Tel: +44(0)20 7958 4837  Email: pakistan.woman2@lshtm-ctu.org |  |
| UGANDA Dr Sam Ononge  Senior Lecturer  Department of Obstetrics & Gynaecology  Makerere University College of Health Sciences  P.O Box 7072  Kampala, Uganda  Tel: +256-772-486301 |  |
