# Supplementary material for: Tranexamic acid for the prevention of postpartum bleeding in women with anaemia: study protocol for an international, randomised, double-blind, placebo-controlled trial
Source: Trials. 2018 Dec 29;19:712. doi: 10.1186/s13063-018-3081-x (PMC6311062; doi:10.1186/s13063-018-3081-x)
Supplement: Supplementary file 8 — Data Management Committee (DMC) membership. (DOCX 23 kb) [file 13063_2018_3081_MOESM8_ESM.docx]

## Data Monitoring Committee

**Membership:**

| **NAME** | **AFFILIATION** | **EXPERTISE** |
| --- | --- | --- |
| Jane Armitage (Chair) | National Perinatal Epidemiology Unit (NPEU)  Nuffield Department of Population Health  University of Oxford  UK | Professor of Clinical Trials and Epidemiology & Honorary Consultant in Public Health Medicine. |
| Olufemi T. Oladapo | Department of Reproductive Health and Research  World Health Organization  Geneva  Switzerland | Maternal, perinatal, and newborn health research and public health expert. |
| Maria Quigley | National Perinatal Epidemiology Unit (NPEU)  Nuffield Department of Population Health  University of Oxford  UK | Professor of Statistical Epidemiology. |
