# Supplementary material for: Tranexamic acid for the prevention of postpartum bleeding in women with anaemia: study protocol for an international, randomised, double-blind, placebo-controlled trial
Source: Trials. 2018 Dec 29;19:712. doi: 10.1186/s13063-018-3081-x (PMC6311062; doi:10.1186/s13063-018-3081-x)
Supplement: Supplementary file 9 — Trial Steering Committee (TSC) membership. (DOCX 23 kb) [file 13063_2018_3081_MOESM9_ESM.docx]

## Trial Steering Committee

**Membership:**

| **NAME** | **AFFILIATION** | **EXPERTISE** |
| --- | --- | --- |
| France Donnay (Chair) | Tulane School of Public Health and Tropical Medicine, New Orleans. | Adjunct Professor; Independent consultant, with a focus on women’s health policies, programs and practices. |
| Toyin Saraki | Wellbeing Foundation Africa  Nigeria | Founder-President, Wellbeing Foundation Africa; maternal and child health advocate; women’s views representative |
| Tina Lavender | Centre of Global Women’s Health, University of Manchester, UK | Professor of Midwifery |
| Ghazala Mahmud | Fazaia Medical College, Islamabad. Pakistan | Professor of Obstetrics and Gynaecology |
| Ian Roberts | Clinical Trials Unit, London School of Hygiene & Tropical Medicine, UK | Professor of Epidemiology and Public Health; randomised controlled trials; conduct of large scale international trials |
| Haleema Shakur-Still | Clinical Trials Unit, London School of Hygiene & Tropical Medicine, UK | Associate Professor Clinical Trials; randomised controlled trials; conduct of large scale international trials |
| Jens Kieckbusch  (Observer) | Wellcome Trust, 215 Euston Road, London.  NW1 2BE | Grant oversight on behalf of the Funder |
